# Primary Osteosarcoma of the Breast: A Rare Case Report and Literature Review

**DOI:** 10.3389/fonc.2022.875793

**Published:** 2022-06-09

**Authors:** Shike Li, Qingfeng Xue, Wenyu Shi

**Affiliations:** ^1^Graduate School, Dalian Medical University, Dalian, China; ^2^Department of Hematology, Affiliated Hospital of Nantong University, Nantong, China; ^3^Department of Oncology, Affiliated Hospital of Nantong University, Nantong, China

**Keywords:** primary osteosarcoma of the breast, extraskeletal osteosarcoma of the breast, extraskeletal osteosarcoma, osteosarcoma, thoracic oncology

## Abstract

**Background:**

Primary osteosarcoma of the breast (POB) is an extremely aggressive and heterogeneous neoplasm that originates from nonepithelial elements of the mammary gland and accounts for fewer than 1% of breast cancers and fewer than 5% of all sarcomas.

**Case Presentation:**

An 83-year-old Chinese woman went to our hospital because of a palpable mass she had had for 8 months in the left breast accompanied by persistent dull pain for 10 days. This mass was initially misdiagnosed as a degenerating fibroadenoma and was graded as probably benign (BI-RADS category 3) by ultrasonography (US) and computed tomography (CT) plain scan and contrast enhancement of chest. Eight months later, it was presumed to be highly malignant and graded as BI-RADS category 4C because of its rapid growth and more calcifications by US and CT. 99mTc-MDP whole-body bone imaging showed that there was a mass-like abnormal radioactive concentration of Tc-99m outside the bone of the left chest. The lumpectomy of the left breast was indicated, and the pathological findings were POB. She succumbed to respiratory failure caused by multiple lung metastases 4 months after the operation.

**Conclusion:**

POB is rare, and US and CT cannot reliably distinguish the causes of calcified breast masses between benign and malignant tumors. It can be diagnosed by pathology when metaplastic carcinoma, malignant phyllodes tumor, or carcinosarcoma containing osteoid and bone is excluded. This case could help clinicians to improve the prognosis and treatment of this disease.

## Introduction

Primary osteosarcoma of the breast (POB) accounts for fewer than 1% of all primary malignancies of the breast (1). There are less than 200 cases published *via* case reports or small case series. The exact mechanism of tumorigenesis of POB remains unknown. Studies suggest that it may occur because of neoplastic transformation in a pre-existing breast lesion (including fibroadenoma, phyllodes tumor, or intraductal papilloma), or it may arise from totipotent mesenchymal cells of the breast stroma (2–4). In this article, we report an 83-year-old female diagnosed with POB in the left breast without histories of any breast lesions and radiotherapy. This case could help clinicians to improve the prognosis and treatment of this disease.

## History

An 83-year-old Chinese woman noticed a palpable mass in the left breast that had been there for 8 months. When the woman first came, there was a round mass about 3 cm in size in the inner upper quadrant and the outer upper quadrant of the left breast, without local pain, skin redness, swelling, ulceration, nipple bleeding, or discharge. She had histories of hypertension and diabetes for 7 years and was diagnosed with diabetic nephropathy, chronic kidney disease stage 4, and renal anemia for 3 months, and all the diseases were under treatment and control. She had no family history of tumor disease. Due to the patient’s old age and multiple underlying diseases, no further treatment was performed. Eight months later, she felt a persistent dull pain in her breast for 10 days. The physical examination showed that the left breast was enlarged, and there was a firm, irregular mass, about 7 cm × 8 cm size in the left breast, with unclear boundaries and an acceptable range of motion without other changes in the breast. No mass was found in the right breast, and no enlarged lymph nodes were touched on the bilateral axillary and supraclavicular region.

**Figure 1:**
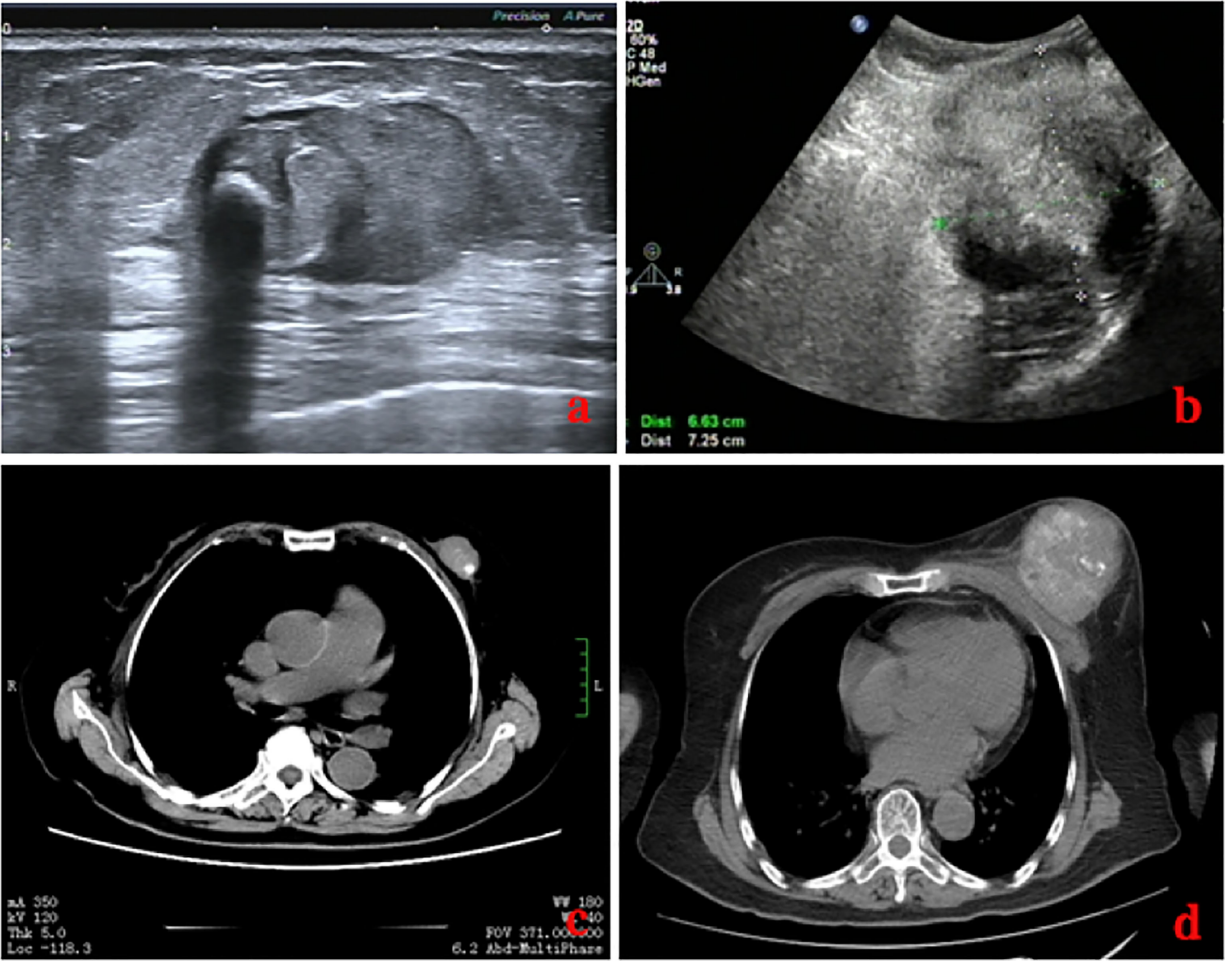
Comparison of images on US **(A, B)** and CT **(C, D)** of the patient before and after 8 months. Eight months later, the lesion was much larger with unclear boundaries, and there were much more calcifications.

**Figure 2:**
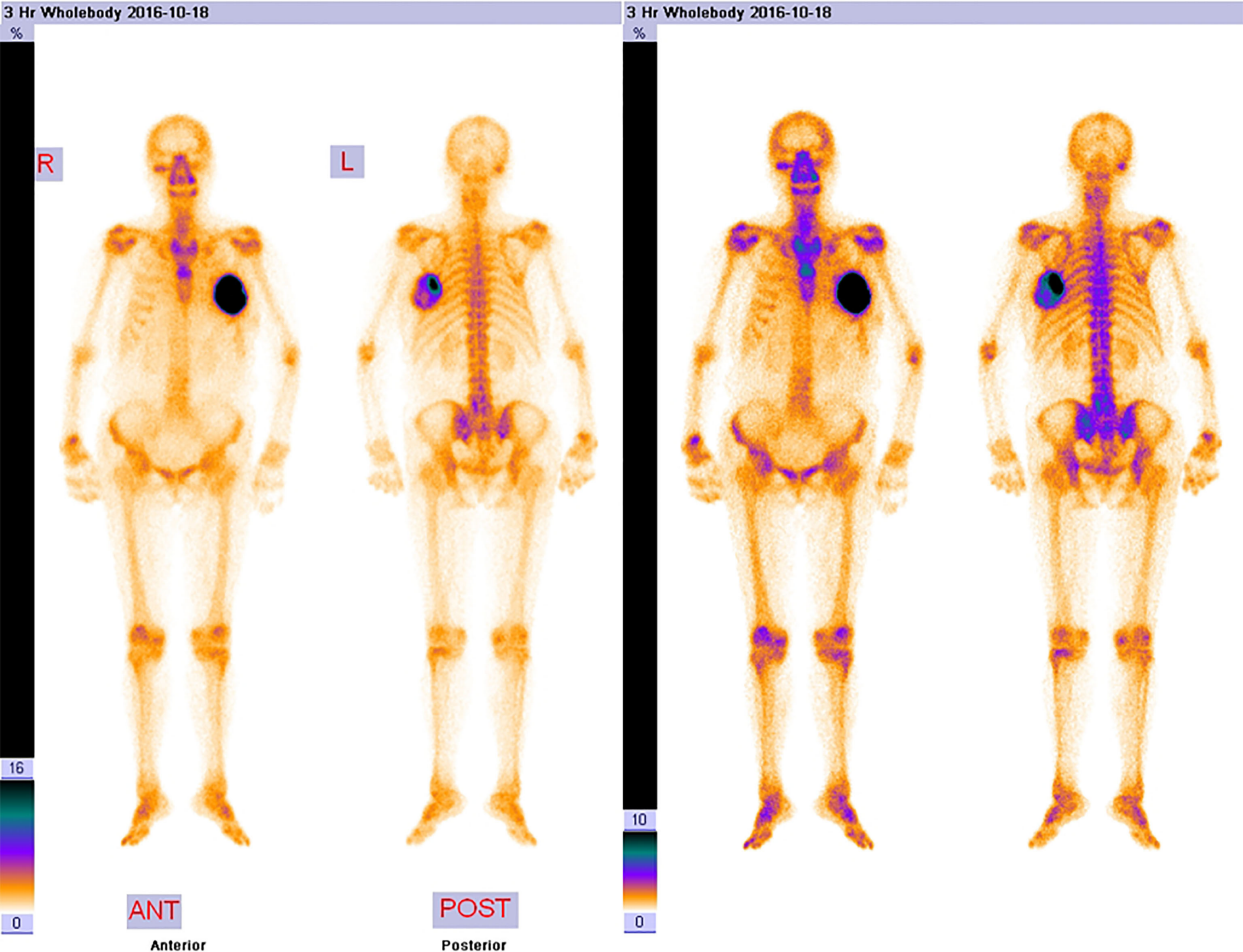
The 99mTc-MDP whole-body bone imaging. It showed that there was a mass-like abnormal radioactive concentration of Tc-99m outside the bone of the left chest, without other abnormal concentrations.

**Figure 3:**
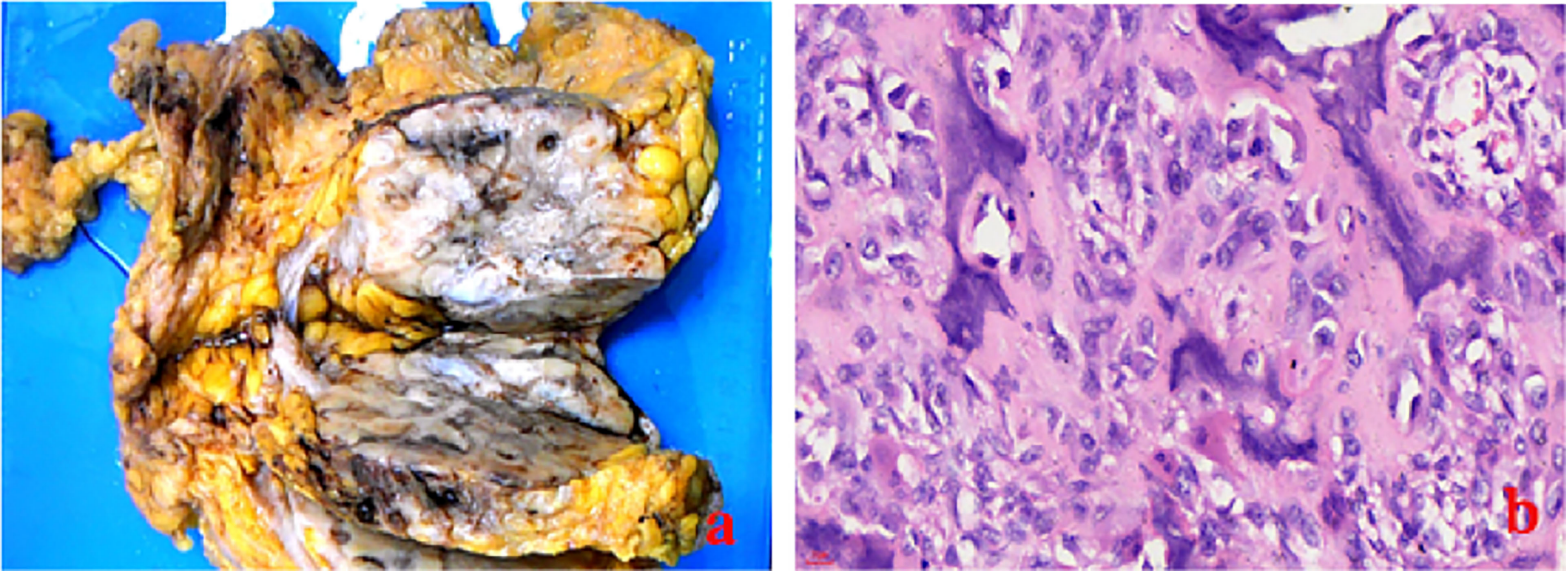
The gross specimen **(A)** and the histologic result (**B**:ross specimen **(A)** showed a 7 cm × 8 cm × 8 cm mass in the central region with clear boundary, regular shape, and pectoralis major invaded, and the histologic result (**B**: HE×40) showed that it was rich in osteoid tissues, heteromorphic osteoblast-like cells, and multinucleated giant cells.

**Figure 4:**
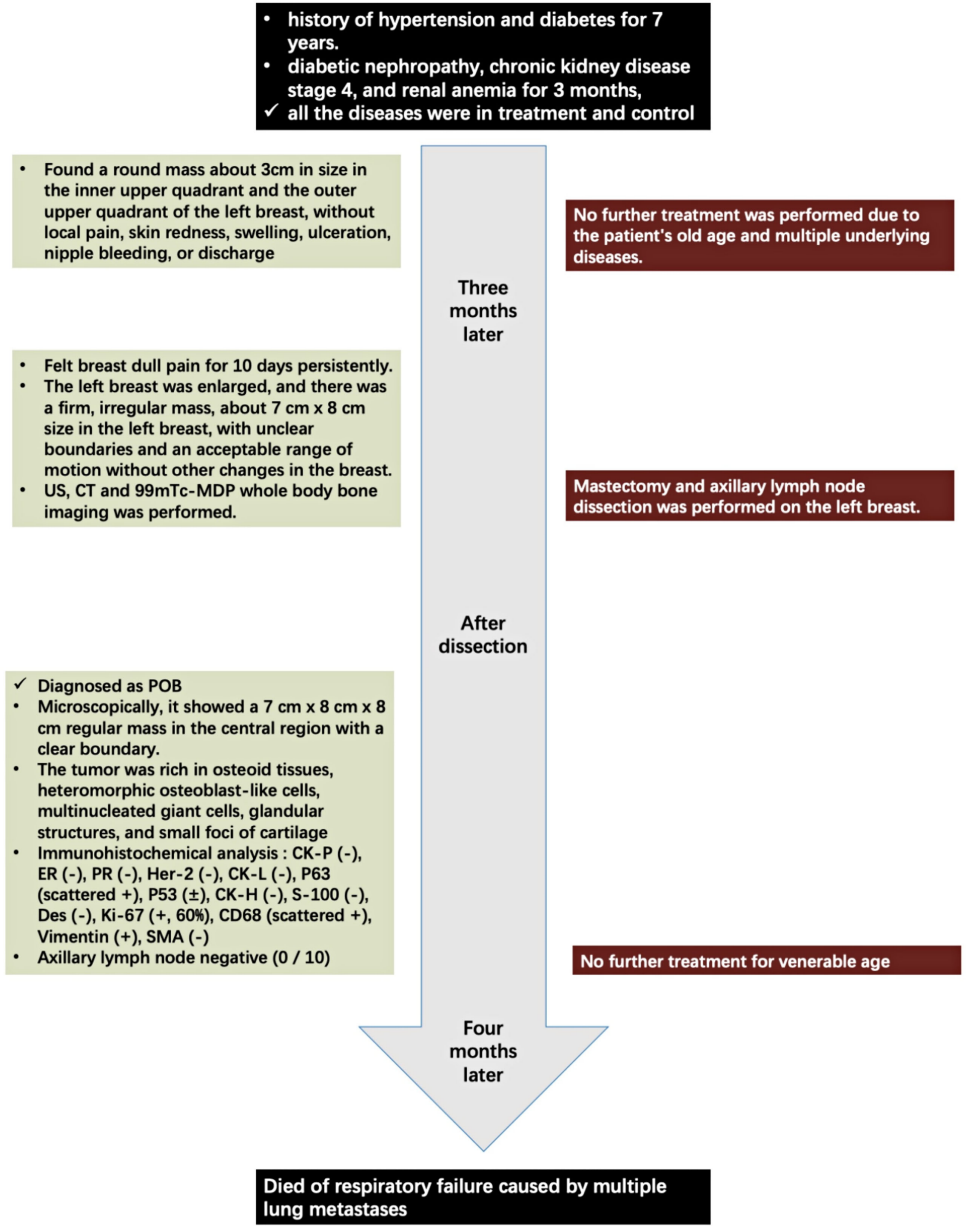
Timeline.

The laboratory tests showed that hemoglobin was 76 g/L, alkaline phosphatase was 554 U/L, and the serum tumor markers were all in a normal range. A calcified mass was found at the first examination of ultrasonography (US) and computed tomography (CT) plain scan and contrast enhancement of chest (**Figure 1**). The lesion was considered to be a degenerative fibroadenoma and was classified as a benign tumor according to Breast Imaging and Reporting Data System (BI-RADS) category 3. After 8 months, it was presumed to be highly malignant and graded as BI-RADS category 4C because of its rapid growth and more calcifications at the examination of US and CT plain scan and contrast enhancement of chest (**Figure 1**). Biopsy was performed according to the patient’s preference, resulting in specimens showing to be a malignant phyllodes tumor of the breast with ossification or osteosarcoma. Then, 99mTc-MDP whole-body bone imaging was performed, and it showed that there was a mass-like abnormal radioactive concentration of Tc-99m outside the bone of the left chest, without other abnormal concentrations (**Figure 2**). Mastectomy and axillary lymph node dissection was performed on the left breast, and the histological results were observed (**Figure 3**). Microscopically, it showed a 7 cm × 8 cm × 8 cm regular mass in the central region with a clear boundary—the pectoralis major was invaded. The tumor was rich in osteoid tissues, heteromorphic osteoblast-like cells, multinucleated giant cells, and small foci of cartilage, which is consistent with osteosarcoma. Immunohistochemical analysis showed Special AT-rich-binding protein 2 (SATB2) (+), Vimentin (+), CK-Pan (AE1/AE3) (−), CK-Low Molecular Weight (CK-LMW) (−), CK-High Molecular Weight (CK-HMW) (−), CK56 (−), CK14 (−), P63 (scattered +), P53 (±), S-100 (−), desmin (des) (−), Smooth Muscle Actin (SMA) (−), Cam5.2 (−), CD10 (−), CD34 (−), CD117(−), CD68 (scattered +), Estrogen receptor (ER) (−), progesterone receptor (PR) (−), Human Epidermal growth factor Receptor 2 (Her-2) (−), and Ki-67 (+, 60%). Axillary lymph node was negative (0/10). After the operation, she did not have any further treatment because of her venerable age. Four months later, she died of respiratory failure caused by multiple lung metastases (**Figure 4**).

## Discussion

POB is a rare, invasive, and heterogeneous tumor. Mammary sarcomas are rarely seen and compose <1% of all primary breast malignancies (5). The most common breast sarcomas include fibrosarcoma, malignant fibrous histiocytoma, angiosarcoma, and liposarcoma, accounting for 12.5% of breast sarcomas (6). The origin of the tumor is uncertain. Researchers approach this tumor similar to a sarcoma (cells originated from totipotent mesenchymal cells of the mammary gland or mesodermal remnants), while Emad A. Rakha showed that almost all POBS are derived from an epithelial origin after being under metaplastic transformation (7).

At present, there are less than 200 cases of POB reported. Compared with traditional skeletal osteosarcoma, POB is more common in elderly patients (60–80 years old) (3, 5). Yet, a case reported a diagnosis of POB in a male patient (4) and a 16-year-old young female patient. The etiology of POB remains unclear. It is reported that it may be related to breast radiation therapy and trauma, professional exposure to vinyl chloride, and artificial implants (8). Nevertheless, the current case did not show these potential underlying triggering factors.

The clinical characteristics of POB are that the tumor is shallow, has multiple nodules, and can be bilateral. It often presents as a rapidly enlarging mass, and a minority (18%) of POB is associated with pain (3). Unlike osteosarcoma, which has high serum alkaline phosphatase (AKP), POB has normal or slightly increased serum AKP level (9), which probably reflects small amounts of neoplastic bone pathologically, and seldom visible matrix mineralization. Our patient has a slightly higher level of AKP, and this level declined following excision.

Imaging examinations of US and CT cannot be used to distinguish between the benign and malignant causes of a calcified breast mass. In the beginning, the mass might be graded as benign and was diagnosed with a calcified fibroadenoma. Nevertheless, the next US and CT considered it as highly malignant and graded as BI-RADS category 4C because of the rapid growth of the mass as well as more calcifications. The differential diagnosis of breast calcified masses with invasive clinical and imaging features is limited (10). It suggests that the characteristics of the calcified lesions differed substantially from the initial visit on imaging examinations when new microcalcifications with fine and irregular shapes surround the initial coarse calcified lesion (11). Furthermore, Tc-99m MDP is a specific marker for osteoid neoplasm and should be used to exclude primary skeletal osteosarcoma. The localization of Tc-99m in extraosseous tumors has been fully confirmed, but its intensity is generally considered to be lower than that in skeletal neoplasms (12). 99mTc-MDP whole-body bone imaging showed intense uptake in the mass outside the bone of the breast, which also suggested a primary extraskeletal sarcoma rather than a primary breast carcinoma (13). Some authors consider that whole-body PET-CT may be helpful to staging and restaging of extraskeletal osteosarcomas (9). In the present case, it showed a mass-like abnormal radioactive concentration of Tc-99m outside the bone of the left chest, without other abnormal concentrations.

POB presents as a highly malignant mesenchymal neoplasm that produces neoplastic osteoid and bone. The final diagnosis of POB can only be established when an osteogenic sarcoma arising from the underlying bones is excluded and immunohistochemical tests show lack of epithelial differentiation (14). The main histological typing and differential diagnosis of POB is metaplastic carcinoma, malignant phyllodes tumor, or carcinosarcoma containing osteoid and bone. Metaplastic carcinoma always shows an immune response to epithelial markers, while POB lacks markers of epithelial differentiation. In this case, antibodies against epithelial markers such as AE1/AE3, Cam5.2, CK56, and CK14 were all negative, evidencing the non-epithelial character of the tumor. Breast malignant phyllodes tumor is a hyperplasia of both breast stroma and epithelium. Spindle cells in tumor were positive to varying degrees for Vimentin, SMA, CD34, CD10, and Bcl2, and epithelium markers were usually positive. Here, immunohistochemical results sustaining mesolobe tissue origin such as des, SMA, Vimentin, S100, CD34, CD10, and CD117 were negative; thus, malignant phyllodes tumor is excluded. SATB2 is a sensitive and specific marker for osteoblast differentiation. It is mainly expressed in benign and malignant bone tumors containing osteoblastic differentiation as well as soft tissue tumors with heterogenic bone differentiation. As there was neither chondroid differentiation nor evidence of phylloid tumor or metaplasia in this case, the final diagnosis was POB. Sometimes POB needs to be identified with breast primary cutaneous secretory carcinoma (15).

Due to the rarity of the disease, the optimal treatment strategy for POB varies. Wide excision with negative margins of POB is necessary. Axillary lymph node dissection is not recommended because POB is locally aggressive and metastasizes hematogenously rather than through lymphatics. It is reported that 20 patients with POB underwent axillary lymph node dissection and there were no lymph node metastases (16). The benefit of adjuvant radiotherapy and chemotherapy in terms of overall survival advantage has not been determined (17). As osteosarcoma is resistant to radiation therapy, postoperative radiotherapy is still controversial. Radiotherapy may be performed in cases of positive margin, or a huge mass that cannot be well excised (18). Some studies prompt that adjuvant chemotherapy may be valuable for POB treatment strategies. Adjuvant chemotherapy may be used with high-grade tumors >5 cm and/or positive margins (19, 20). A retrospective study in 266 patients with extraskeletal osteosarcoma showed higher survival rates in patients who received perioperative chemotherapy (21). In the literature, metastases are most common to the lung followed by bone and liver (18). In this case, the patient had multiple lung metastases 4 months after the operation and died of respiratory failure in the end.

As POB is highly aggressive with early recurrence and propensity for hematogenous spread; it has a very poor prognosis. It is reported that the 5-year overall survival (OS) probability was 38%, and the 10-year OS rate was 32% (3). Twenty-four cases of POB were reported in the literature; the 1-year, 2-year, and 5-year OS rates were 78.7%, 68.1%, and 52.5%, respectively; and the 1-year OS rate of eight highly differentiated patients was 100%, compared with 10 poorly differentiated patients, whose 1-year OS rate was only 5% (16). As incisional margin involvement is an important predictor for local disease recurrence, the size of the tumor, number of mitoses, and existence of stromal atypia are other prognostic indicators (22).

As there is limited evidence to guide treatment, further research is needed to explore the treatment of this invasive disease.

## Conclusion

POB is an extremely rare disease with a poor prognosis. The origin of the disease is still obscure. Breast scan of US and CT often shows dense calcification mass, which is initially misdiagnosed as a benign tumor. The final diagnosis is based on a comprehensive IHC panel and meticulous pathological examination. The main histological typing and differential diagnosis of POB is metaplastic carcinoma or carcinosarcoma containing osteoid and bone. Till now, the best treatment for POB has been an adequate extent of surgery, whereas the role of adjuvant chemoradiotherapy remains unclear. Further research regarding the mechanism of tumor development, imaging findings, and effective treatment methods is required to improve the survival rate of POB.

## Data Availability Statement

The original contributions presented in the study are included in the article/**Supplementary Material**. Further inquiries can be directed to the corresponding author.

## Ethics Statement

Written informed consent was obtained from the individual(s) for the publication of any potentially identifiable images or data included in this article.

## Author Contributions

Guarantor of integrity of the entire case study: SL, QX, and WS. Study concepts and design: SL and WS. Literature research: All authors. Imaging and data analysis: SL and WS. Manuscript preparation: All authors. All authors contributed to the article and approved the submitted version.

## Conflict of Interest

The authors declare that the research was conducted in the absence of any commercial or financial relationships that could be construed as a potential conflict of interest.

## Publisher’s Note

All claims expressed in this article are solely those of the authors and do not necessarily represent those of their affiliated organizations, or those of the publisher, the editors and the reviewers. Any product that may be evaluated in this article, or claim that may be made by its manufacturer, is not guaranteed or endorsed by the publisher.

## References

[1] Burk KS Fang Y Porembka JH. Diagnostic Evaluation and Treatment of Primary Breast Osteosarcoma: A Case Report. Breast J (2019) 25(4):709–11. doi: 10.1111/tbj.13305 31077474

[2] Ali AK Hamoud AM Faleh AM Bin SAA Halldor B. Primary Osteosarcoma of the Breast Arising in an Intraductal Papilloma. Case Rep Radiol (2017) 2017:5787829. doi: 10.1155/2017/5787829 28713607 PMC5497655

[3] Silver SA Tavassoli FA. Primary Osteogenic Sarcoma of the Breast: A Clinicopathologic Analysis of 50 Cases. Am J Surg Pathol (1998) 22(8):925–33. doi: 10.1097/00000478-199808000-00002 9706972

[4] Mujtaba B Nassar SM Aslam R Garg N Madewell JE Taher A. Primary Osteosarcoma of the Breast: Pathophysiology and Imaging Review. Curr Problems Diagn Radiol (2020) 49(2):116–23. doi: 10.1067/j.cpradiol.2019.01.001 30655112

[5] Dey S Chaudhury MK Basu SK Manna AK Dutta SK. Primary Osteosarcoma of Breast, a Rare Case. J Clin Diagn Res JCDR (2013) 7(8):1710–1. doi: 10.7860/JCDR/2013/5700.3263 PMC3782943 24086886

[6] Bahrami A Resetkova E Ro JY Ibañez JD Ayala AG. Primary Osteosarcoma of the Breast: Report of 2 Cases. Arch Pathol Lab Med (2007) 131(5):792–5. doi: 10.1043/1543-2165(2007)131[792:POOTBR]2.0.CO;2 17488168

[7] Omranipour R Ensani F Hassanesfahani M. Primary Breast Osteosarcoma; a Case Report and Review of the Literature. Clin Case Rep (2021) 9(11):e05044. doi: 10.22541/au.162276602.20240137/v1 34815871 PMC8593882

[8] Voutsadakis IA Zaman K Leyvraz S. Breast Sarcomas: Current and Future Perspectives. Breast (2011) 20(3):199–204. doi: 10.1016/j.breast.2011.02.016 21398126

[9] Gao ZH Yin JQ Liu DW Meng QF Li JP. Preoperative Easily Misdiagnosed Telangiectatic Osteosarcoma: Clinical–Radiologic–Pathologic Correlations. Cancer Imaging (2013) 13(4):520–6. doi: 10.1102/1470-7330.2013.0042 PMC3864225 24334494

[10] Greenspan A. Benign Bone-Forming Lesions: Osteoma, Osteoid Osteoma, and Osteoblastoma. Skeletal Radiol (1993) 22(7):485–500. doi: 10.1007/BF00209095 8272884

[11] Kurata K Anan K Ishikawa N Koga K Nakano T. A Case of Primary Extraskeletal Osteosarcoma of the Breast. Surg Case Rep (2018) 4(1):121. doi: 10.1186/s40792-018-0530-4 30232644 PMC6146110

[12] Pickhardt PJ Mcdermott M. Intense Uptake of Technetium-99m-Mdp in Primary Breast Adenocarcinoma With Sarcomatoid Metaplasia. J Nucl Med (1997) 38(4):528–30. doi: 10.1097/00004424-199704000-00008 9098196

[13] Krishnamurthy A. Primary Breast Osteosarcoma: A Diagnostic Challenge. Indian J Nucl Med Ijnm Off J Soc Nucl Med India (2015) 30(1):39–41. doi: 10.4103/0972-3919.147534 PMC4290064 25589804

[14] Kerkar BP Garima D. Primary Osteosarcoma of the Breast. Indian J Of Surg Oncol (2018) 9(4):578–80. doi: 10.1007/s13193-018-0787-x PMC6265182 30538392

[15] Kastnerova L Luzar B Goto K Grishakov V Gatalica Z Kamarachev J. Secretory Carcinoma of the Skin: Report of 6 Cases, Including a Case With a Novel Nfix-Pkn1 Translocation. Am J Surg Pathol (2019) 43(8):1092–8. doi: 10.1097/PAS.0000000000001261 31045890

[16] Khan S Griffiths EA Shah N Ravi S. Primary Osteogenic Sarcoma of the Breast: A Case Report. Cases J (2008) 1(1):148. doi: 10.1186/1757-1626-1-148 18783623 PMC2546375

[17] Momoi H Wada Y Sarumaru S Tamaki N Gomi T Kanaya S. Primary Osteosarcoma of the Breast. Ann Surg Treat Res (2004) 93(4):396–400. doi: 10.1007/BF02968048 15604996

[18] Huang HA Wang SL. Primary Osteosarcoma of the Breast. Formosan J Surg (2021) 54(5):191. doi: 10.4103/fjs.fjs_233_20

[19] Barrow BJ Janjan NA Gutman H Benjamin RS Allen P Romsdahl MM. Role of Radiotherapy in Sarcoma of the Breast – a Retrospective Review of the M.D. Anderson Experience. Radiother Oncol (1999) 52(2):173–8. doi: 10.1111/j.1600-0714.2006.00407.x 10577703

[20] Casali PG Blay JY Bertuzzi A Bielack S Wardelmann E. Bone Sarcomas: Esmo Clinical Practice Guidelines for Diagnosis, Treatment and Follow-Up. Ann Oncol (2014) 25(Suppl 3):113–23. doi: 10.1093/annonc/mdu256 25210081

[21] Longhi A Bielack SS Grimer R Whelan J Windhager R Leithner A. Extraskeletal Osteosarcoma: A European Musculoskeletal Oncology Society Study on 266 Patients. Eur J Cancer (2017) 74:9–16. doi: 10.1016/j.ejca.2016.12.016 28167373

[22] Adem C Reynolds C Ingle JN Nascimento AG. British Journal of Cancer - Primary Breast Sarcoma: Clinicopathologic Series From the Mayo Clinic and Review of the Literature. Br J Cancer (2004). doi: 10.1038/sj.bjc.6601920 PMC2409972 15187996

