# Supplementary figures and images for: Primary Osteosarcoma of the Breast: A Rare Case Report and Literature Review
Source: Front Oncol. 2022 Jun 9;12:875793. doi: 10.3389/fonc.2022.875793 (PMC9218342; doi:10.3389/fonc.2022.875793)

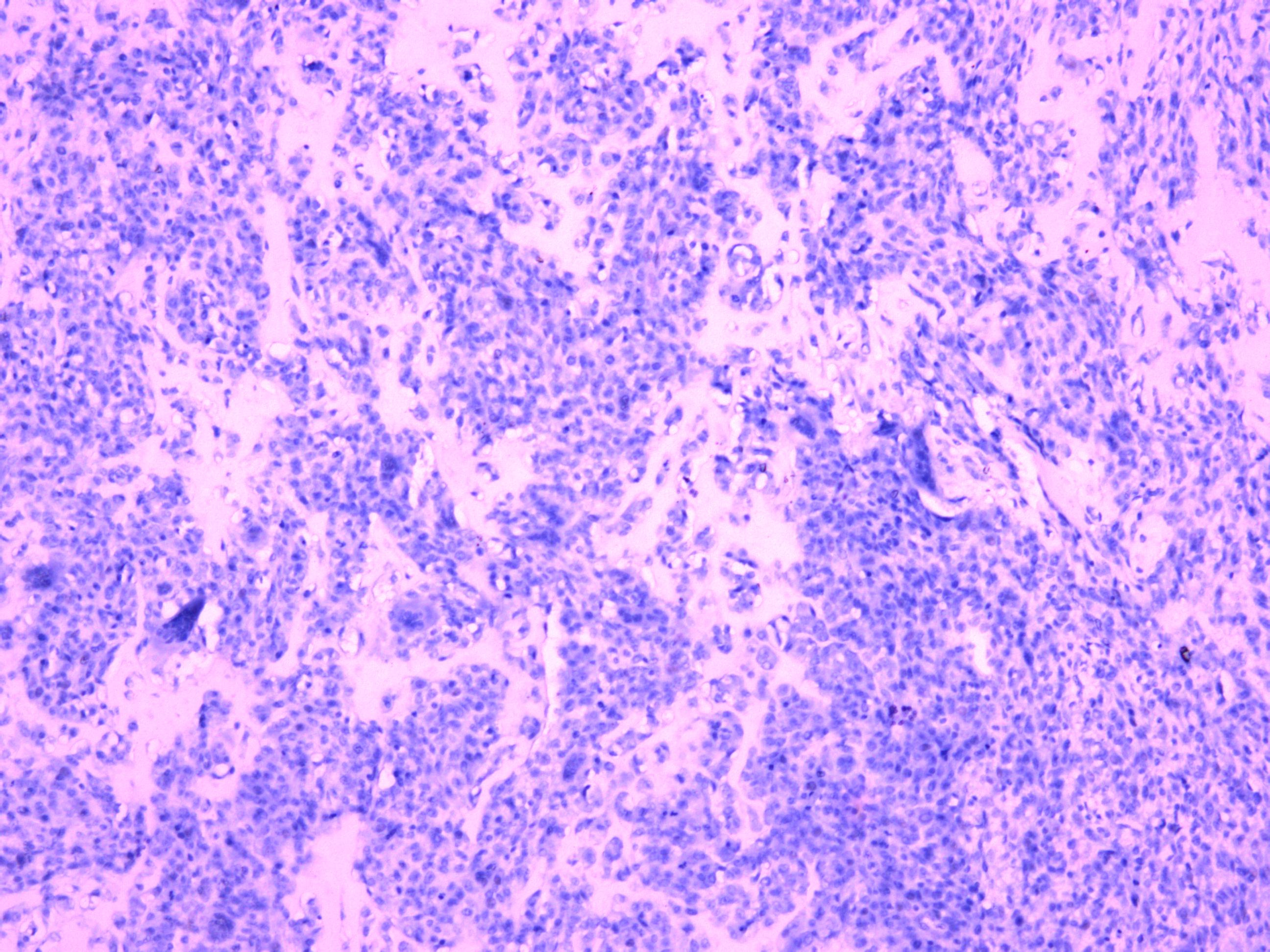

Supplement: Supplementary file 1 [file DataSheet_1.zip › Immunohistochemical analysis/τùàτÉå/CAM5.2 100.JPG]

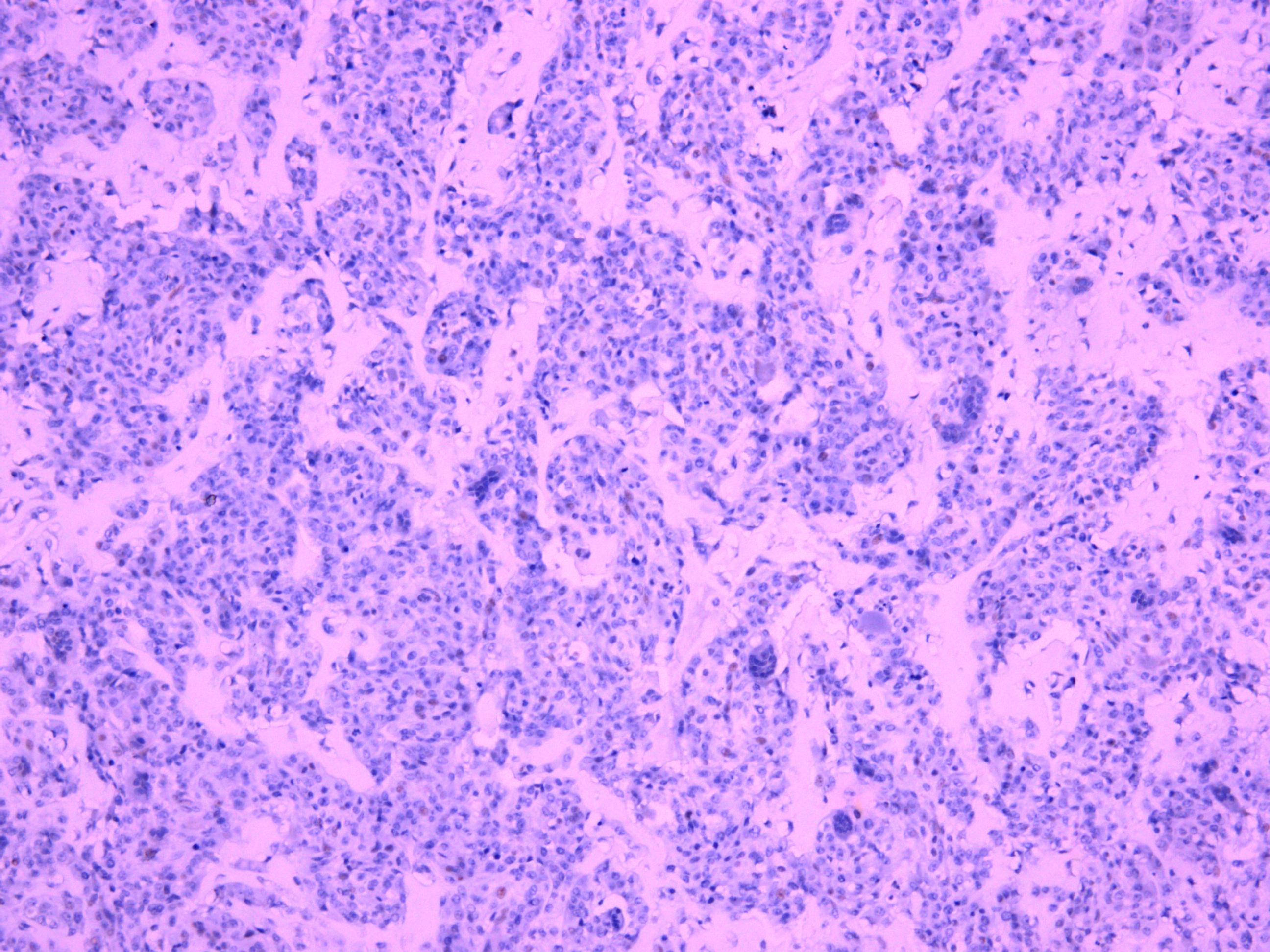

Supplement: Supplementary file 1 [file DataSheet_1.zip › Immunohistochemical analysis/τùàτÉå/CK56 100.JPG]

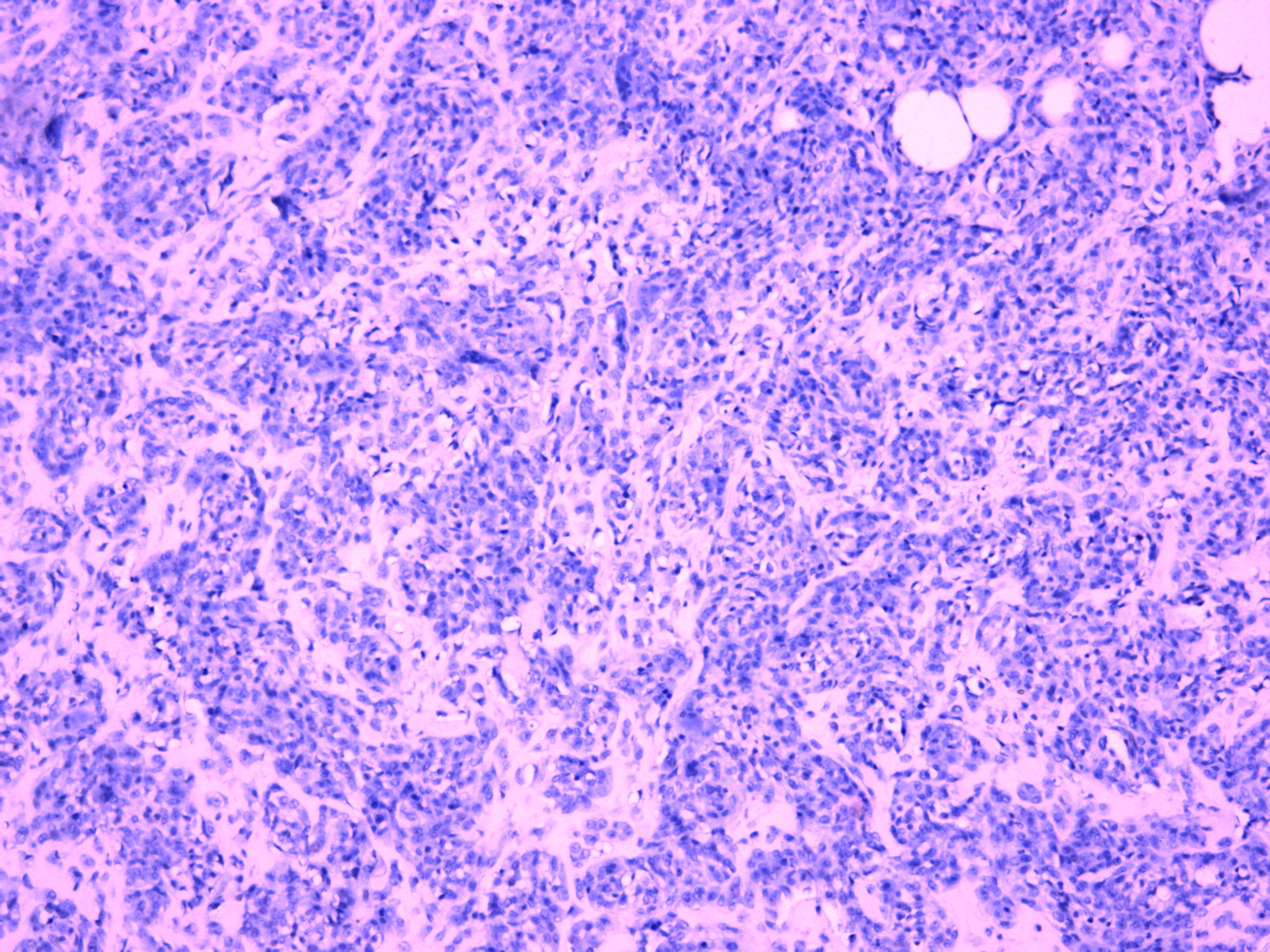

Supplement: Supplementary file 1 [file DataSheet_1.zip › Immunohistochemical analysis/τùàτÉå/CK14 100σÇì.JPG]

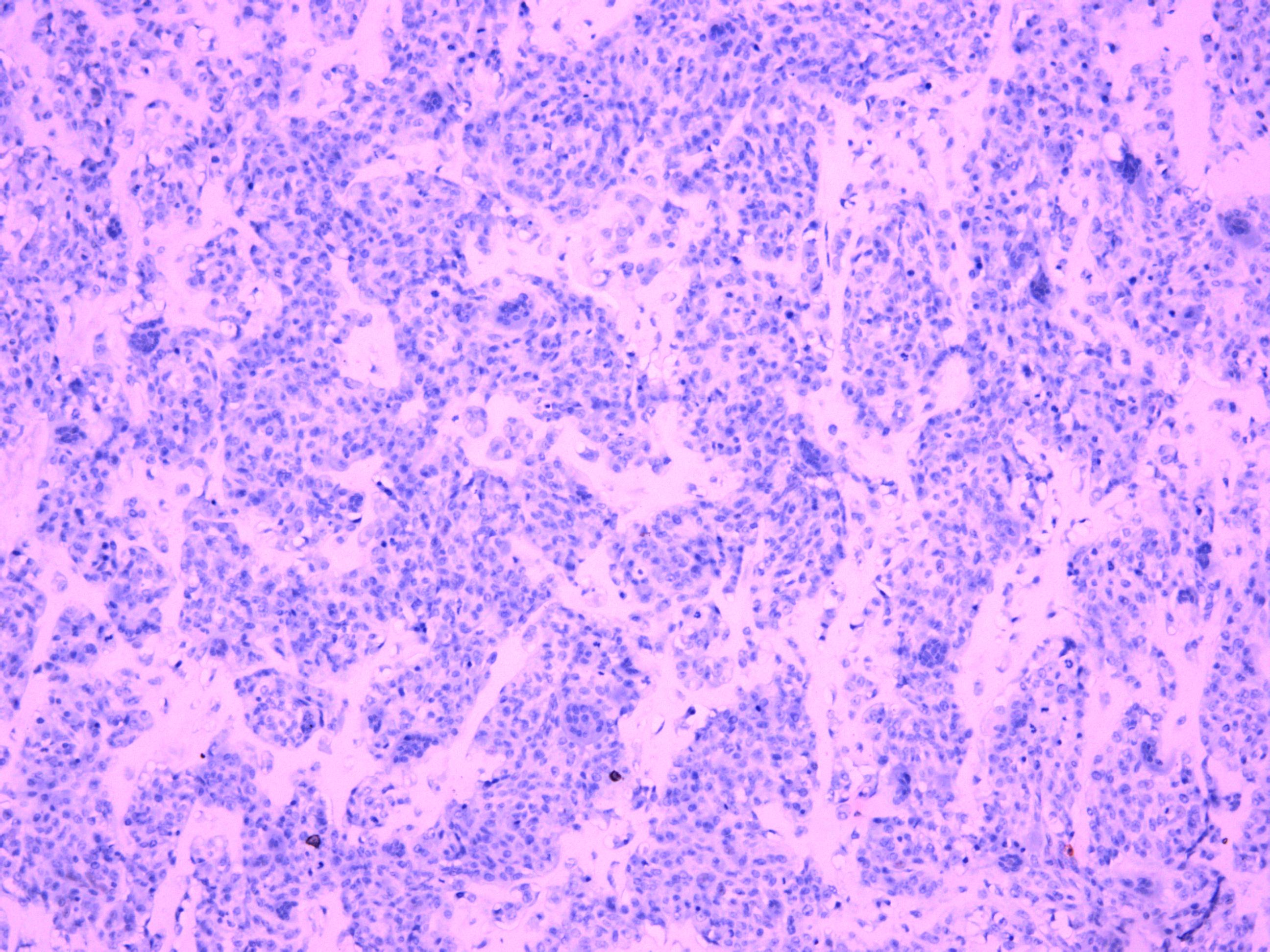

Supplement: Supplementary file 1 [file DataSheet_1.zip › Immunohistochemical analysis/τùàτÉå/CD117 100.JPG]

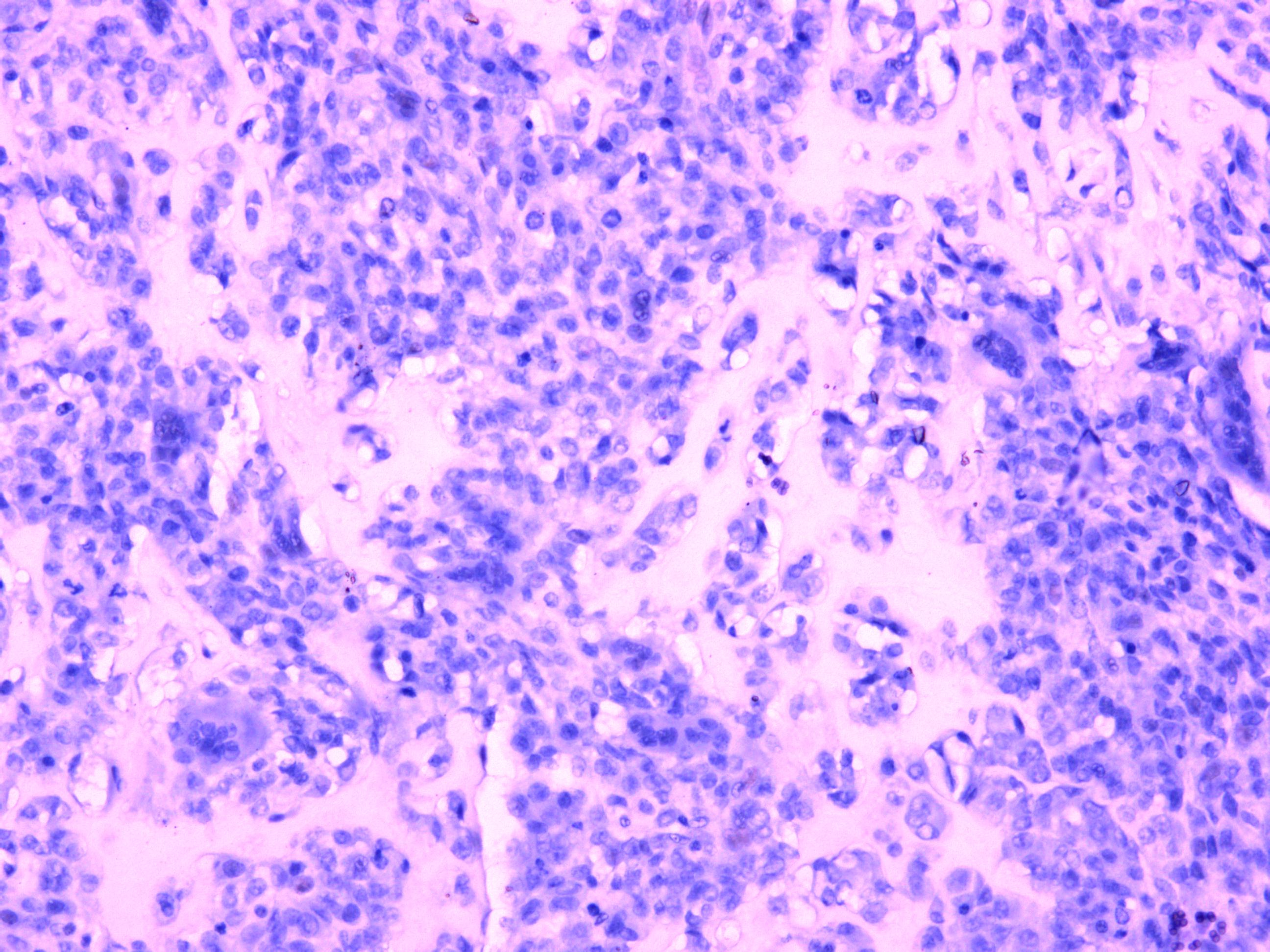

Supplement: Supplementary file 1 [file DataSheet_1.zip › Immunohistochemical analysis/τùàτÉå/CAM5.2 200.JPG]

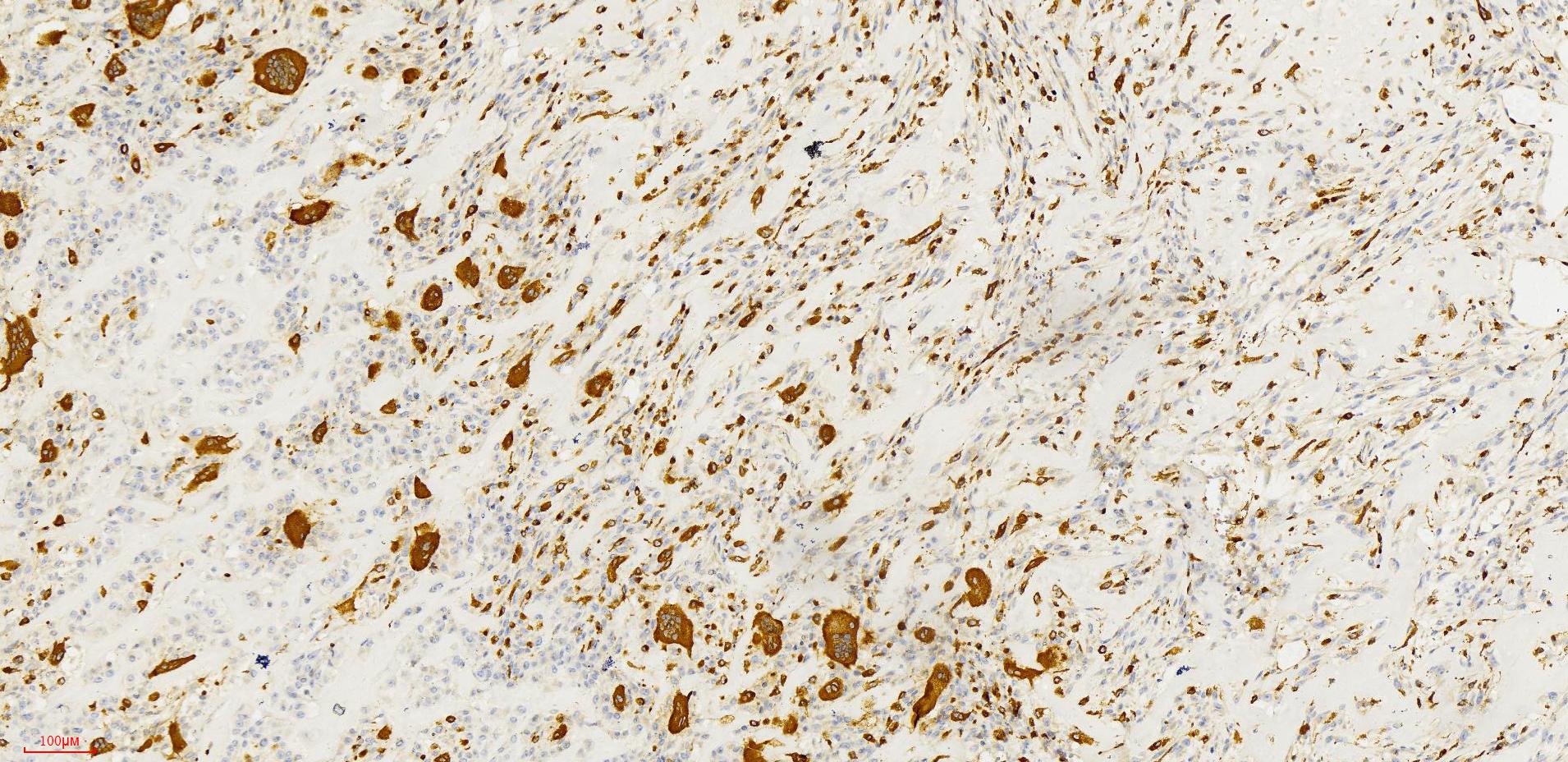

Supplement: Supplementary file 1 [file DataSheet_1.zip › Immunohistochemical analysis/τùàτÉå/cd68 10X.jpg]

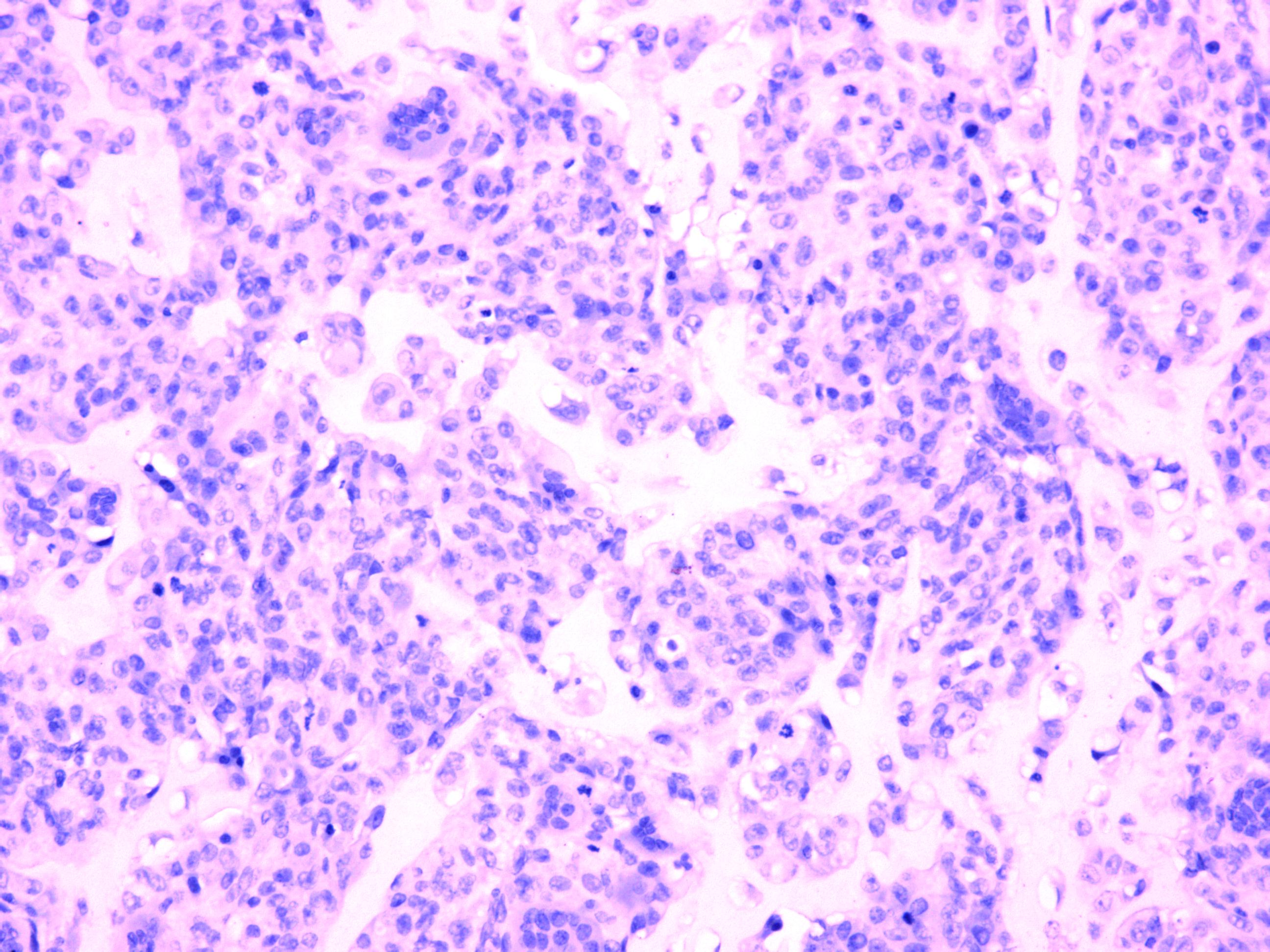

Supplement: Supplementary file 1 [file DataSheet_1.zip › Immunohistochemical analysis/τùàτÉå/CD117 200.JPG]

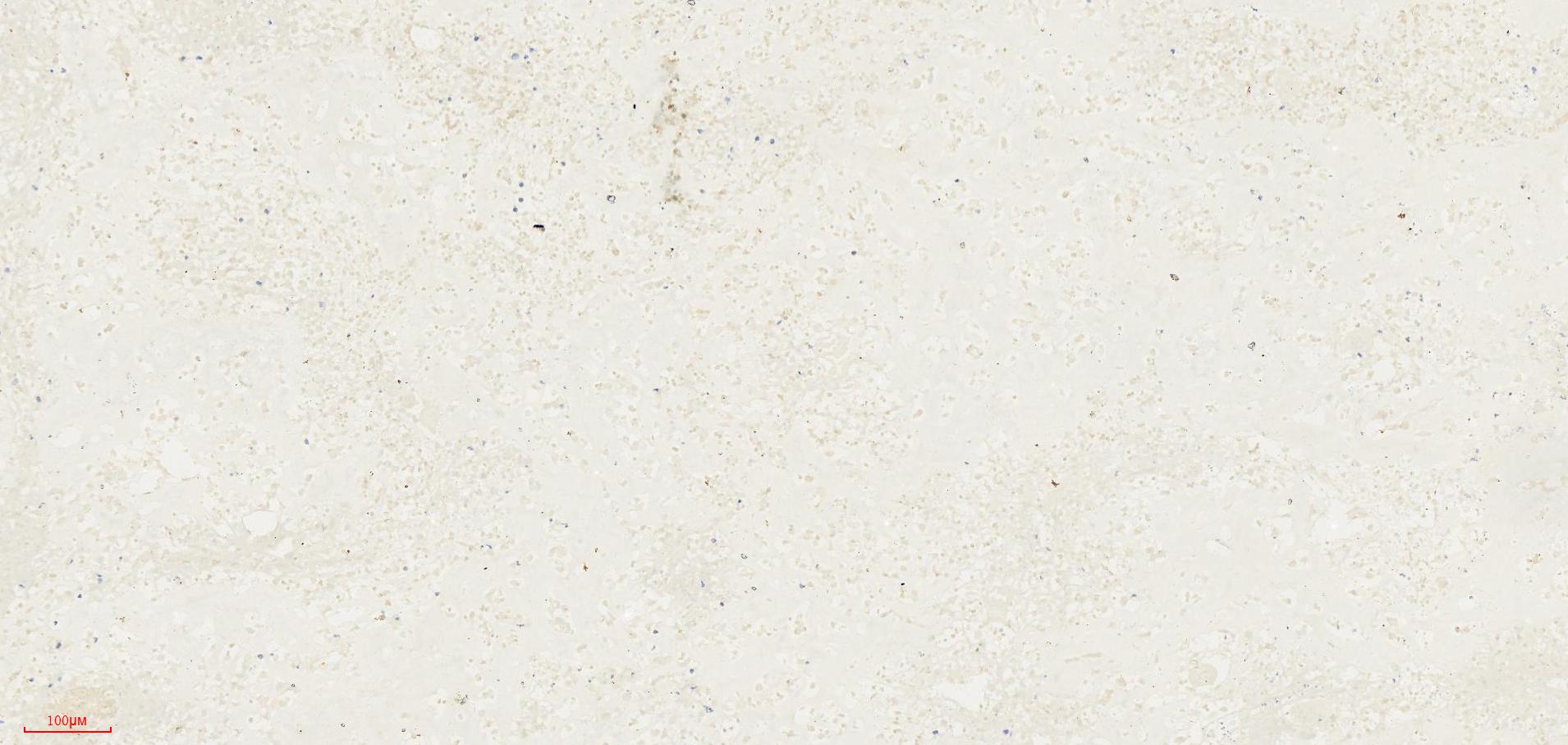

Supplement: Supplementary file 1 [file DataSheet_1.zip › Immunohistochemical analysis/τùàτÉå/her-2 10X.jpg]

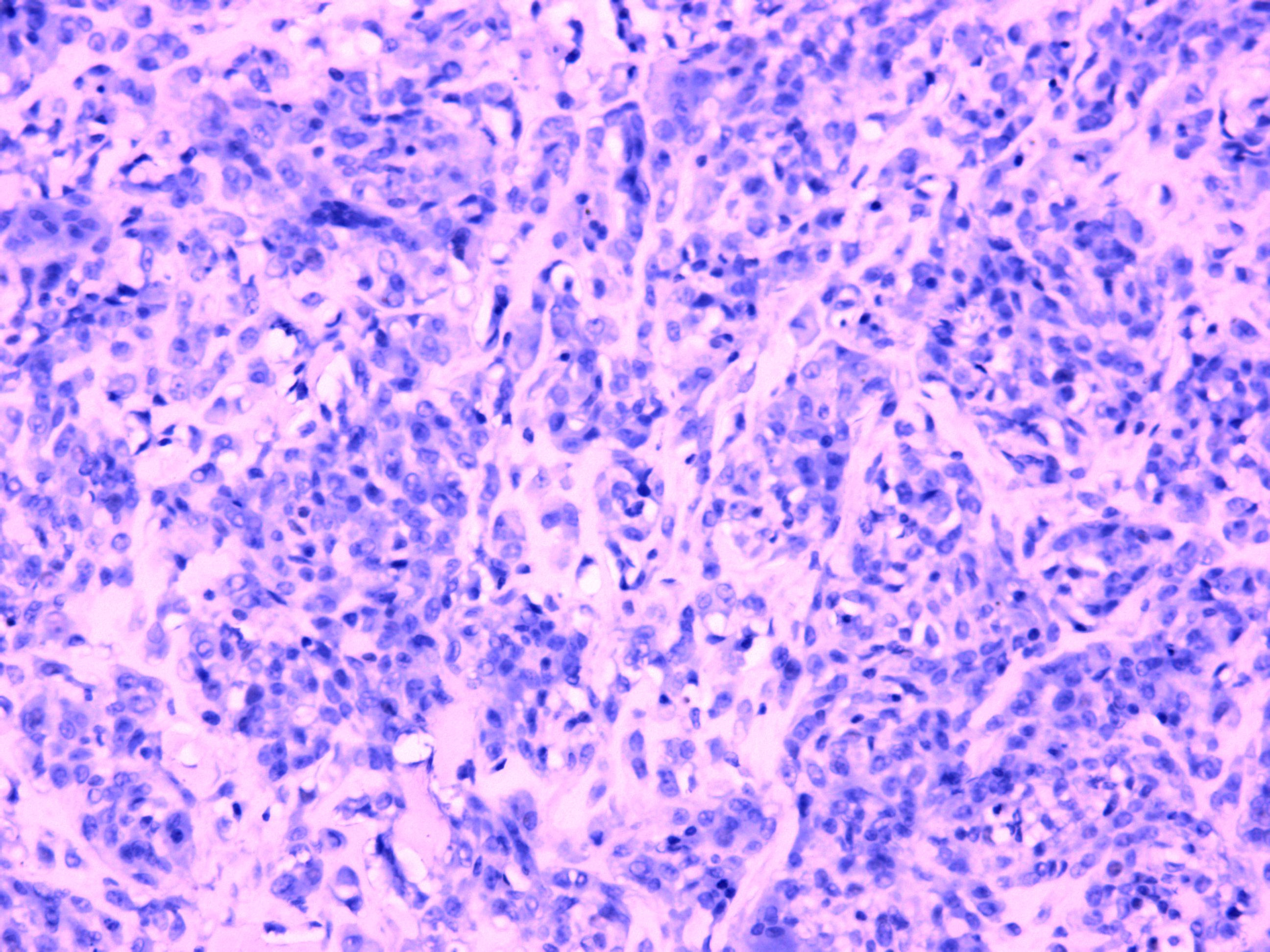

Supplement: Supplementary file 1 [file DataSheet_1.zip › Immunohistochemical analysis/τùàτÉå/CK14 200σÇì.JPG]

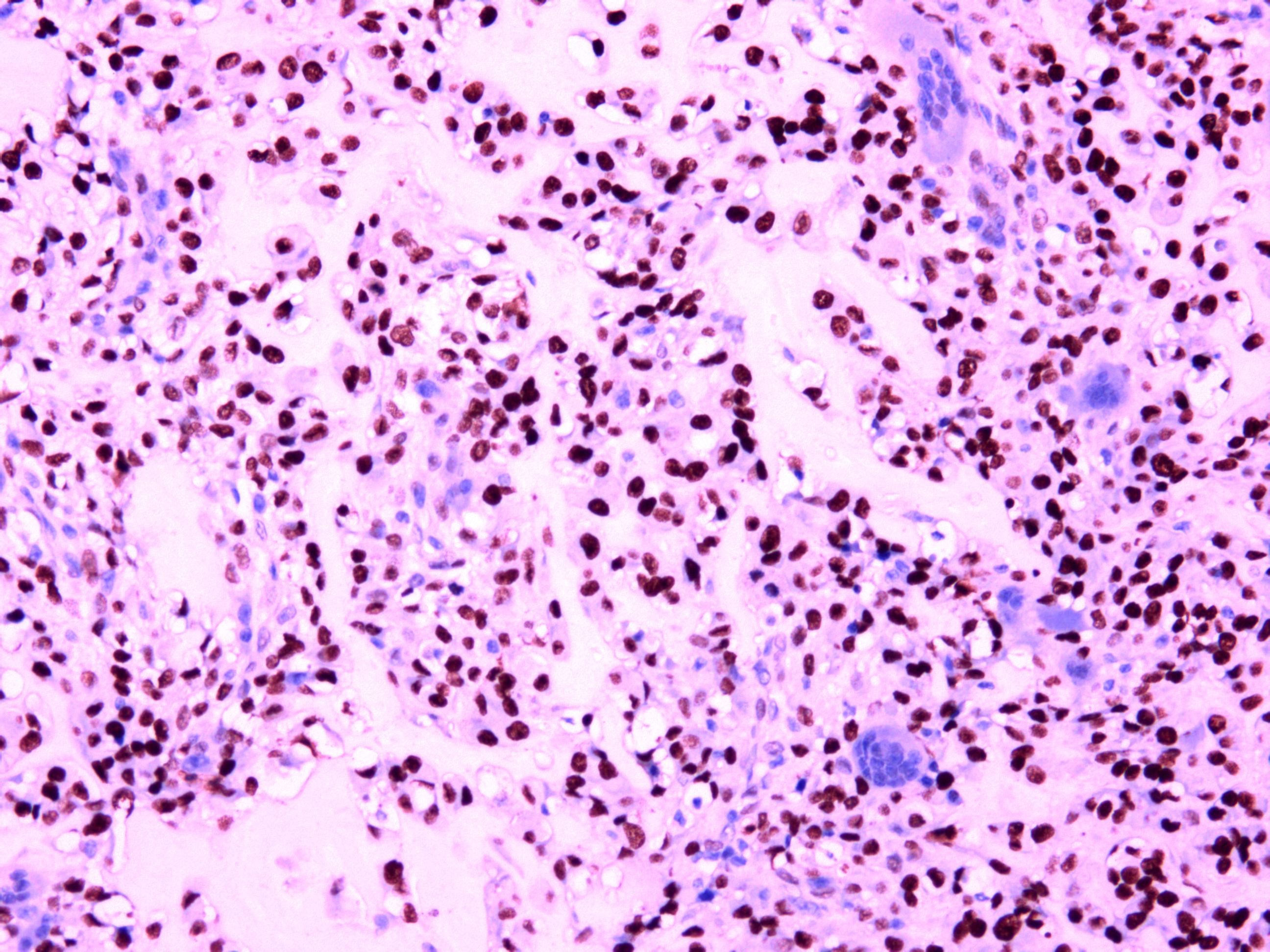

Supplement: Supplementary file 1 [file DataSheet_1.zip › Immunohistochemical analysis/τùàτÉå/SATB2 200-1.JPG]

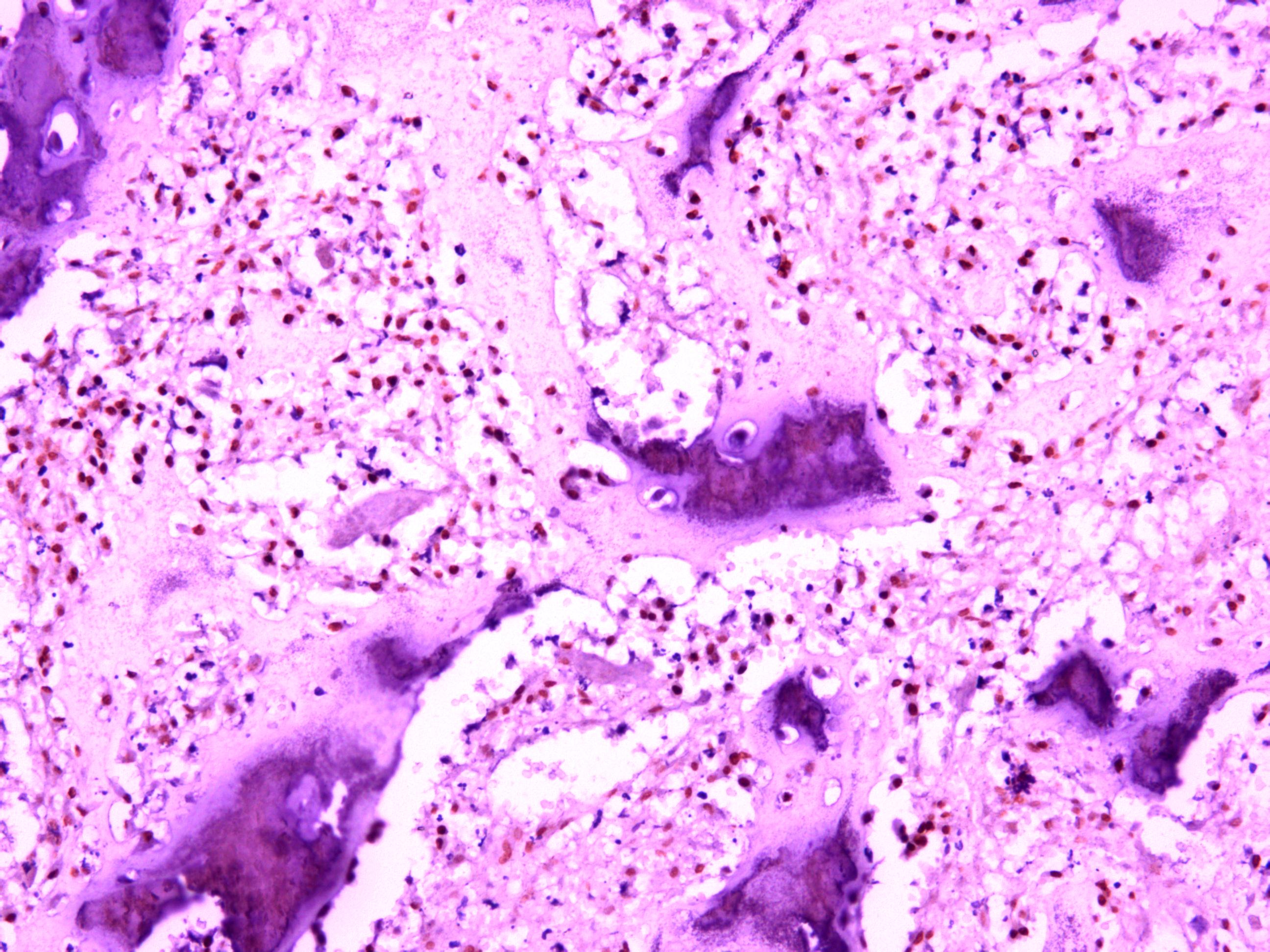

Supplement: Supplementary file 1 [file DataSheet_1.zip › Immunohistochemical analysis/τùàτÉå/SATB2 200-3.JPG]

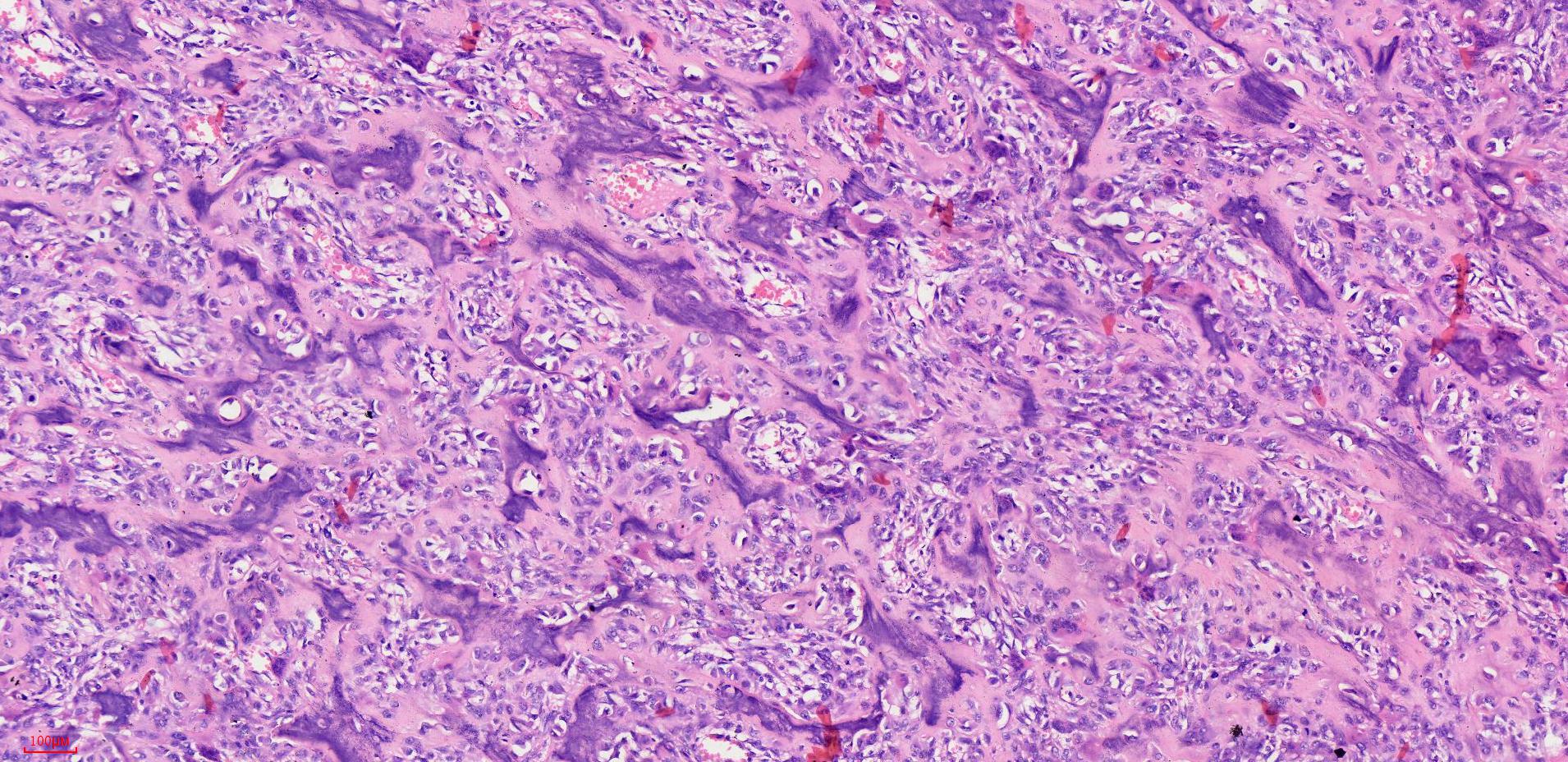

Supplement: Supplementary file 1 [file DataSheet_1.zip › Immunohistochemical analysis/τùàτÉå/HE 10X.jpg]

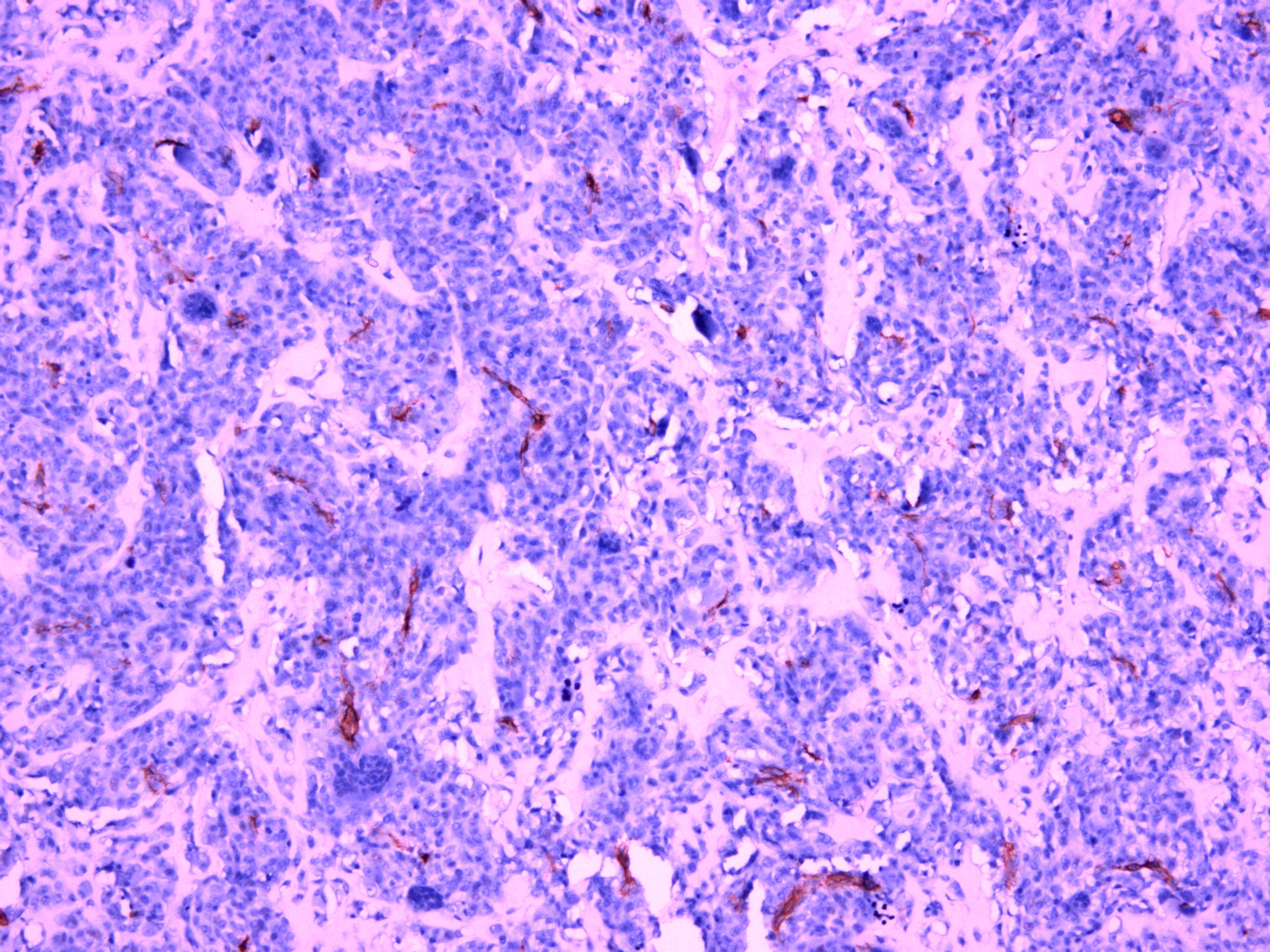

Supplement: Supplementary file 1 [file DataSheet_1.zip › Immunohistochemical analysis/τùàτÉå/CD34 100.JPG]

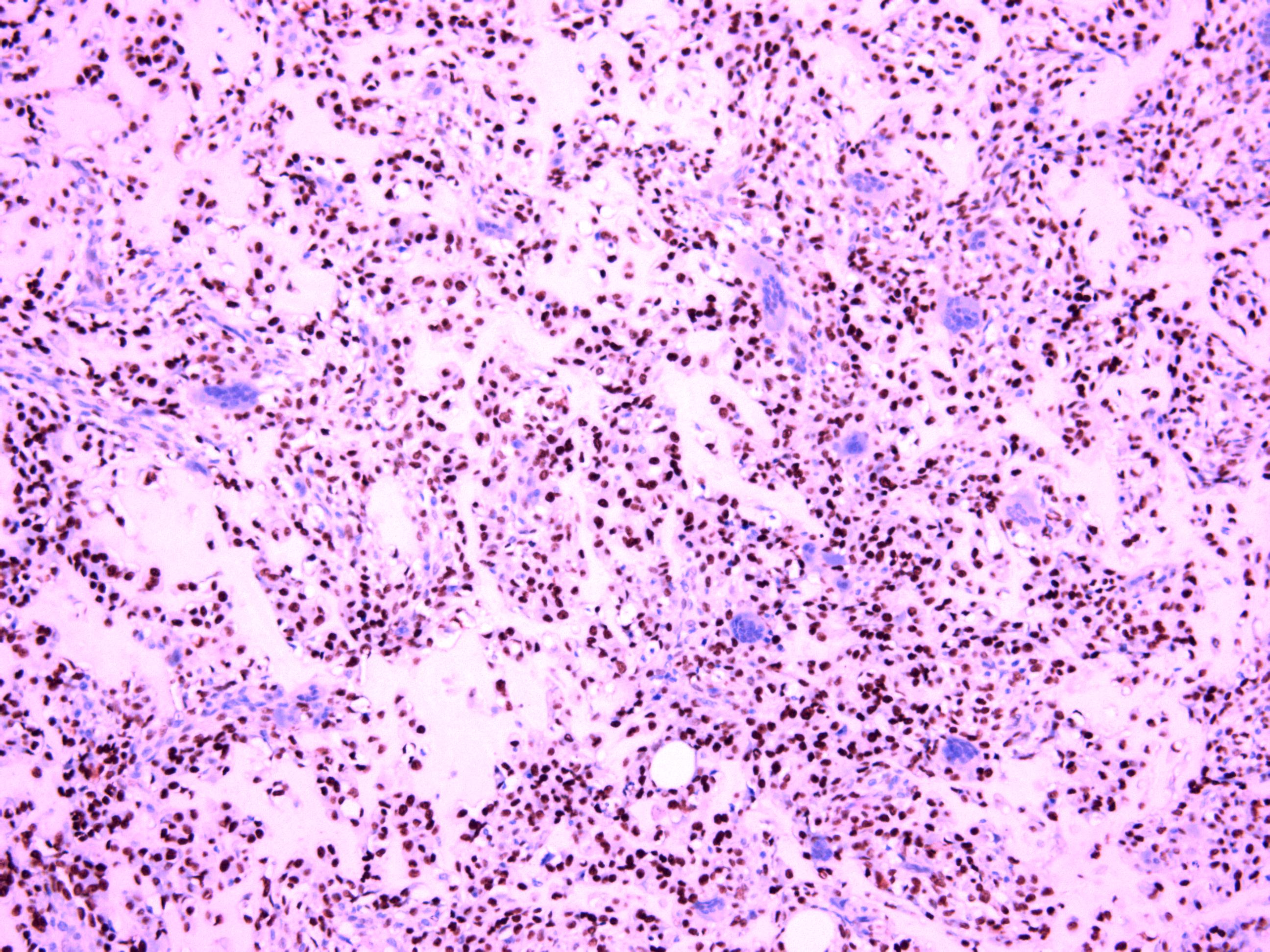

Supplement: Supplementary file 1 [file DataSheet_1.zip › Immunohistochemical analysis/τùàτÉå/SATB2 100.JPG]

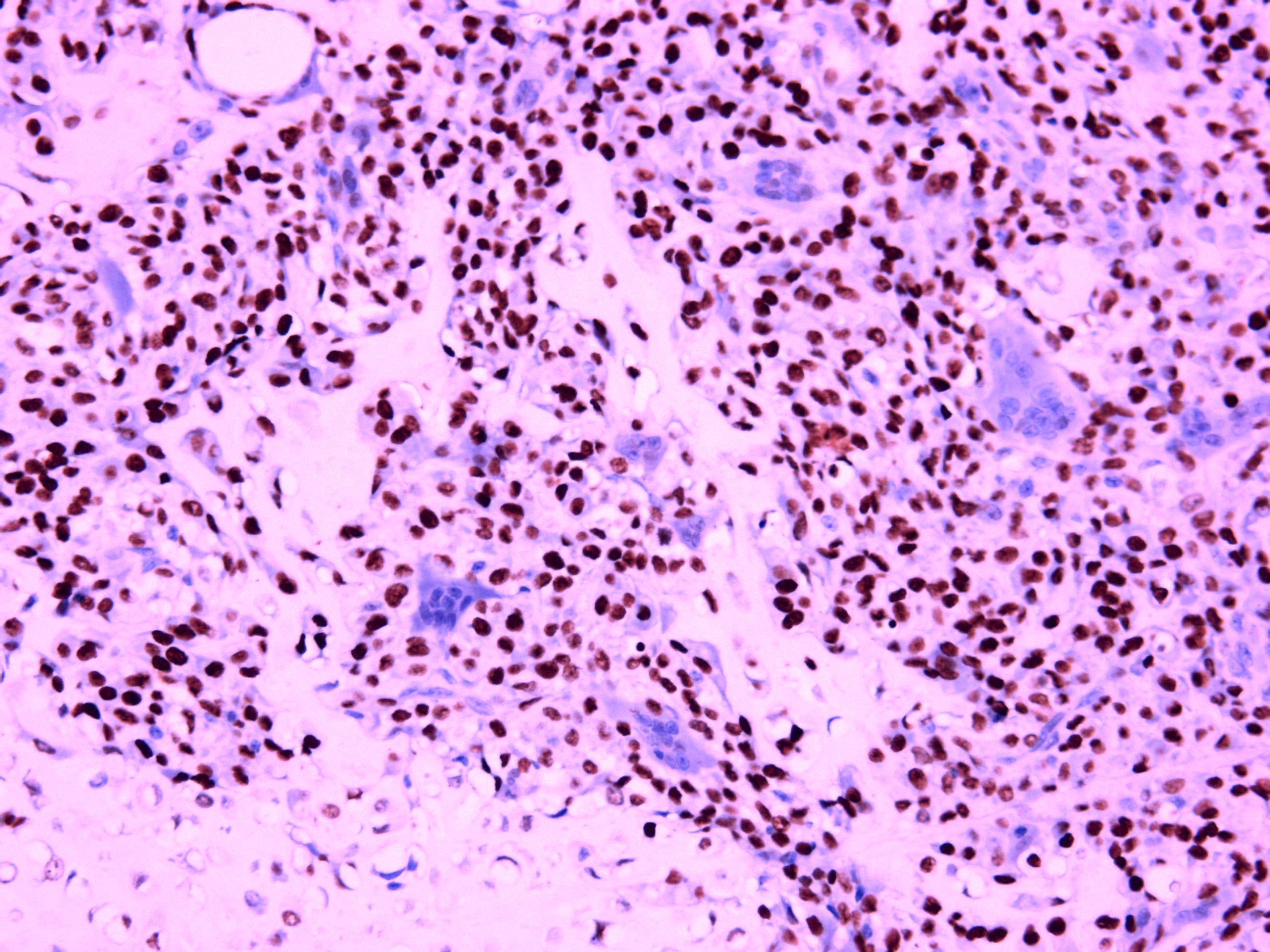

Supplement: Supplementary file 1 [file DataSheet_1.zip › Immunohistochemical analysis/τùàτÉå/SATB2 200-2.JPG]

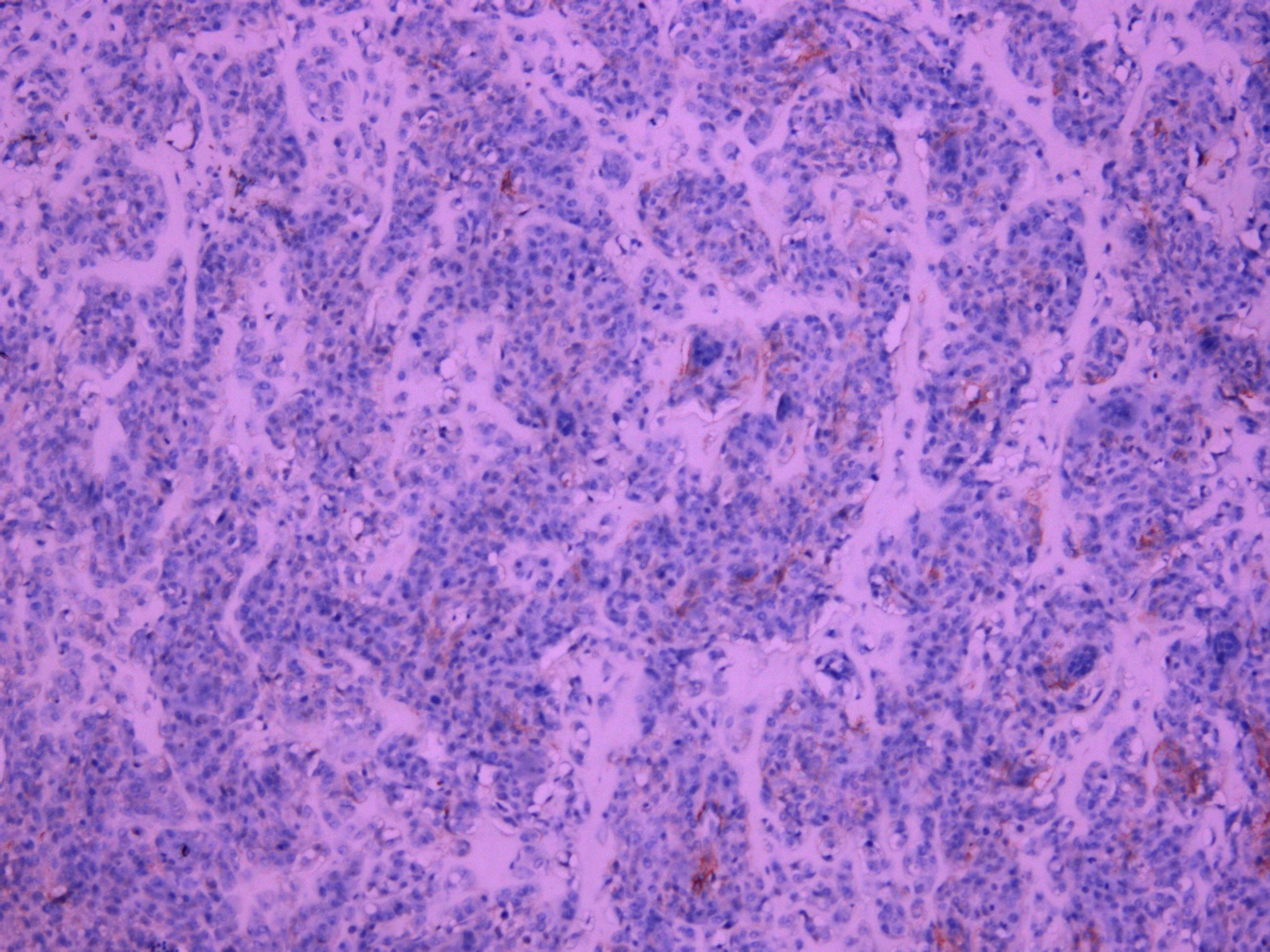

Supplement: Supplementary file 1 [file DataSheet_1.zip › Immunohistochemical analysis/τùàτÉå/CD10 100-2.JPG]

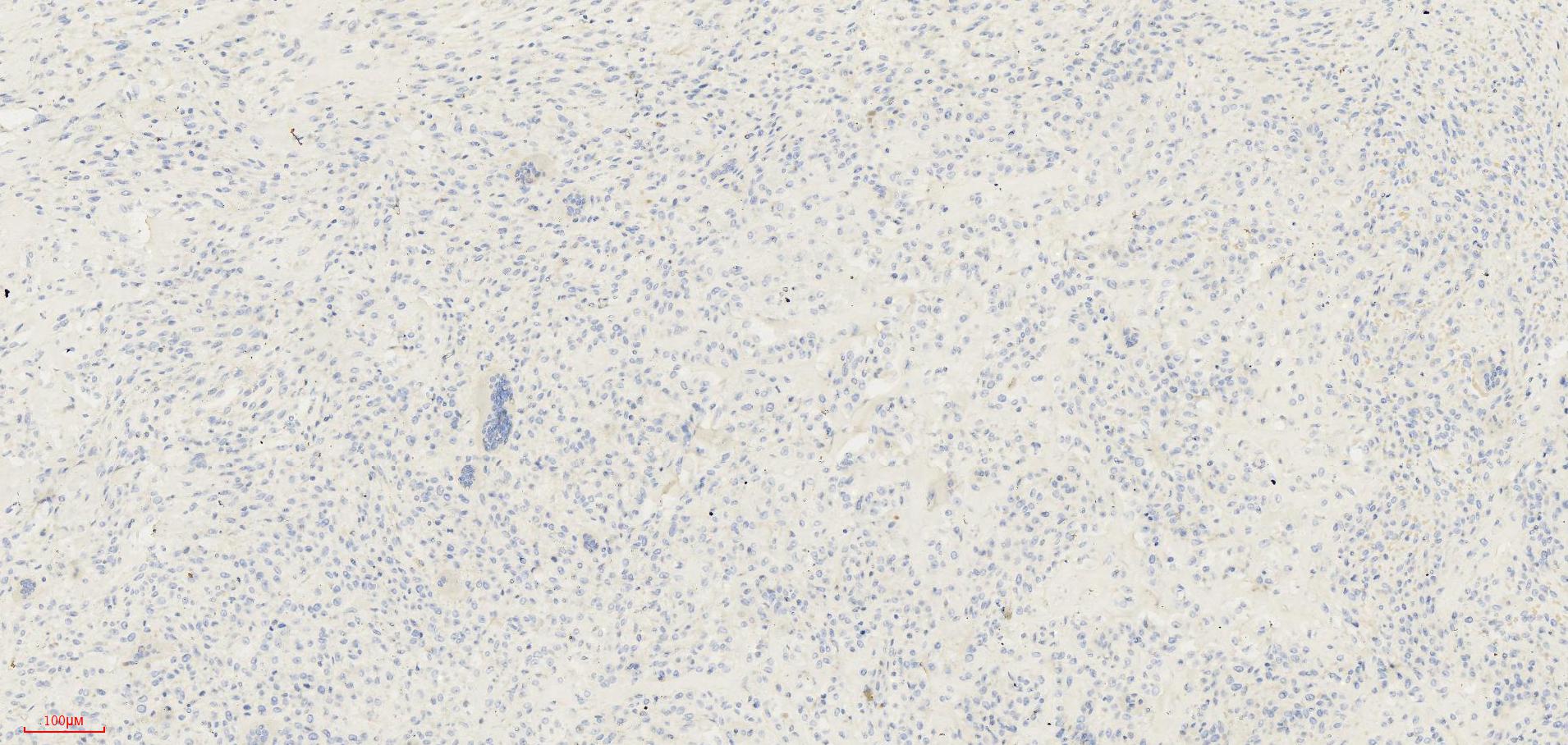

Supplement: Supplementary file 1 [file DataSheet_1.zip › Immunohistochemical analysis/τùàτÉå/des 10X.jpg]

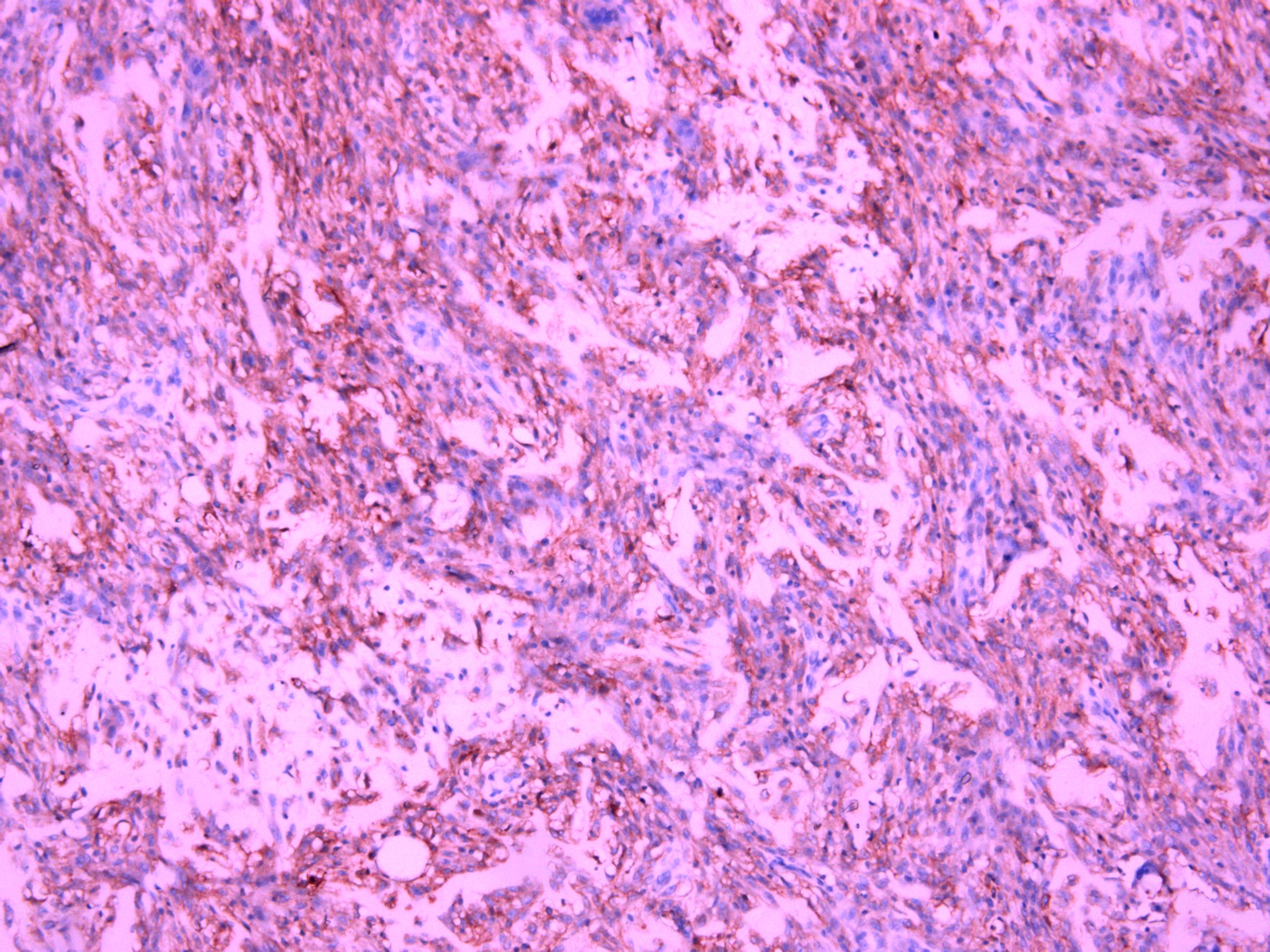

Supplement: Supplementary file 1 [file DataSheet_1.zip › Immunohistochemical analysis/τùàτÉå/CD10 100-1.JPG]

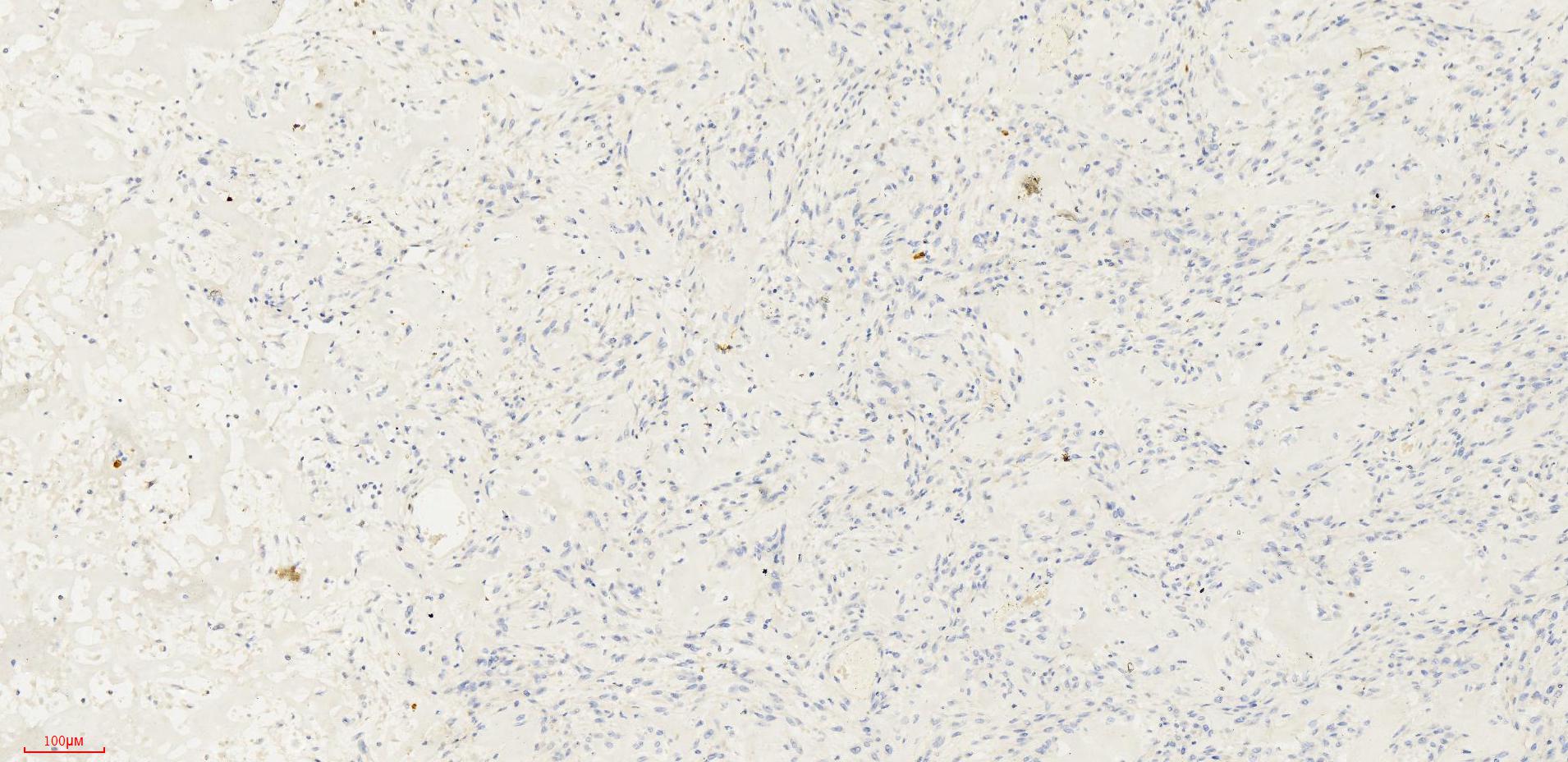

Supplement: Supplementary file 1 [file DataSheet_1.zip › Immunohistochemical analysis/τùàτÉå/s-100 10X.jpg]

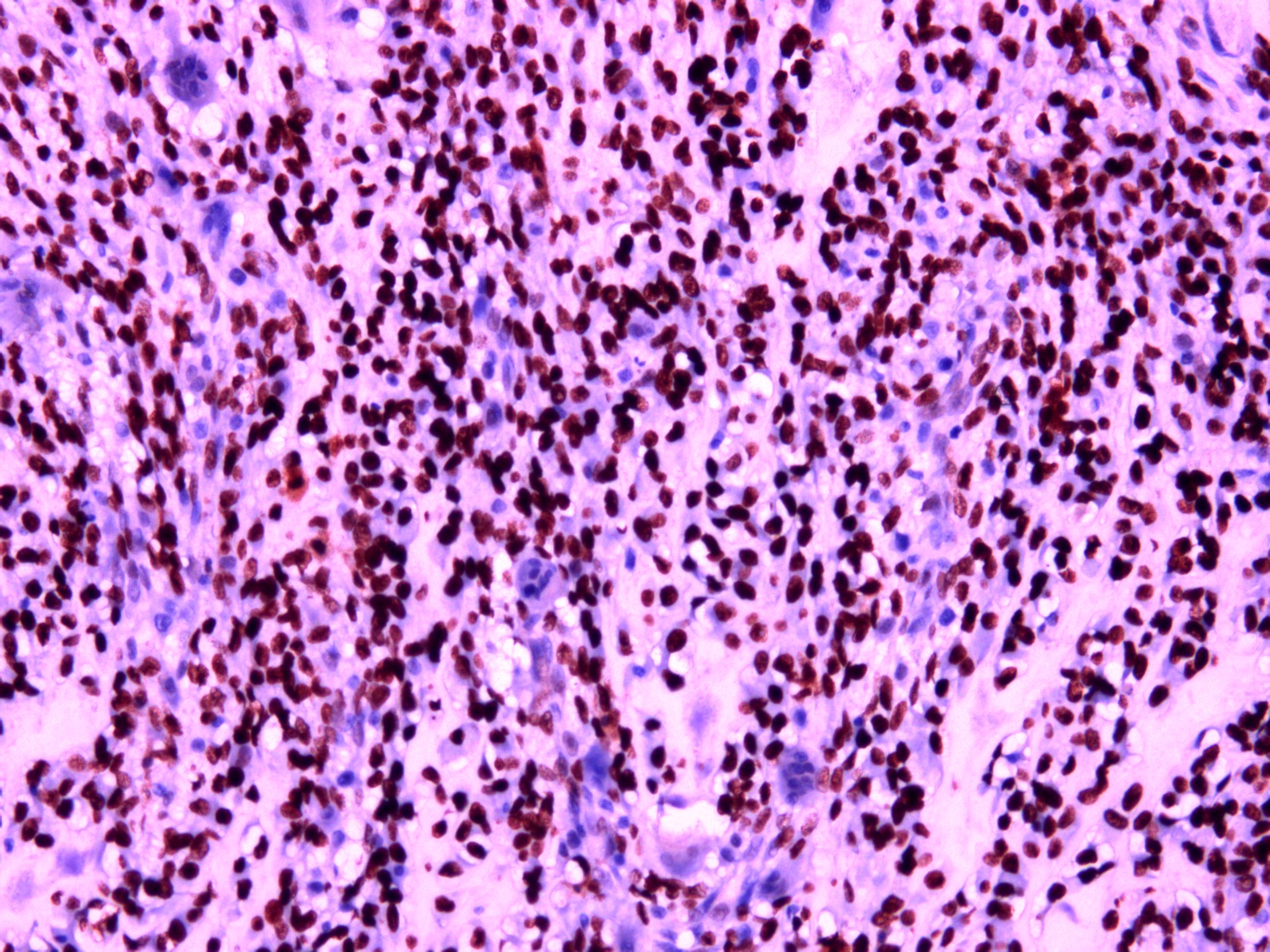

Supplement: Supplementary file 1 [file DataSheet_1.zip › Immunohistochemical analysis/τùàτÉå/SATB2 200-4.JPG]

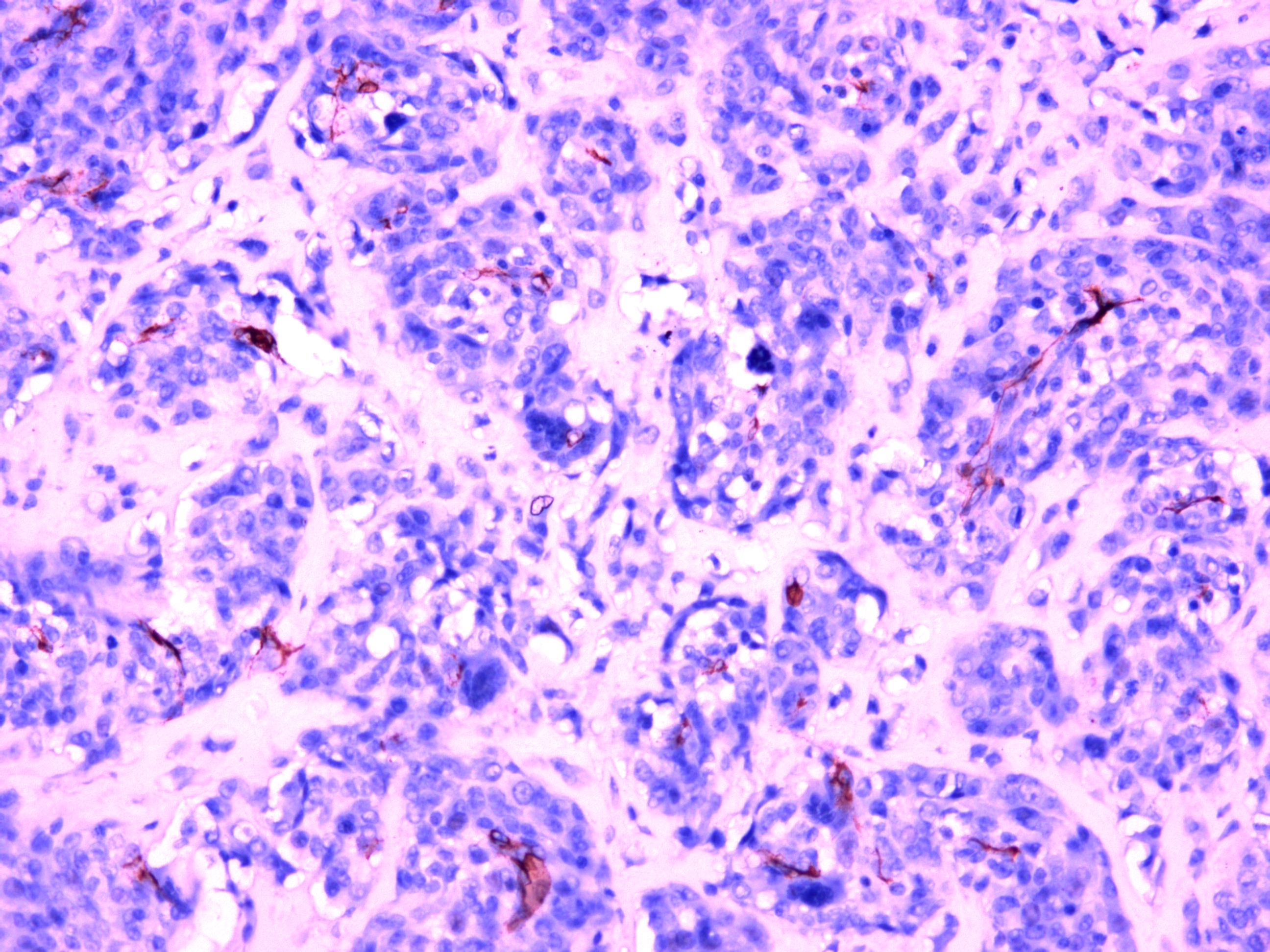

Supplement: Supplementary file 1 [file DataSheet_1.zip › Immunohistochemical analysis/τùàτÉå/CD34 200.JPG]

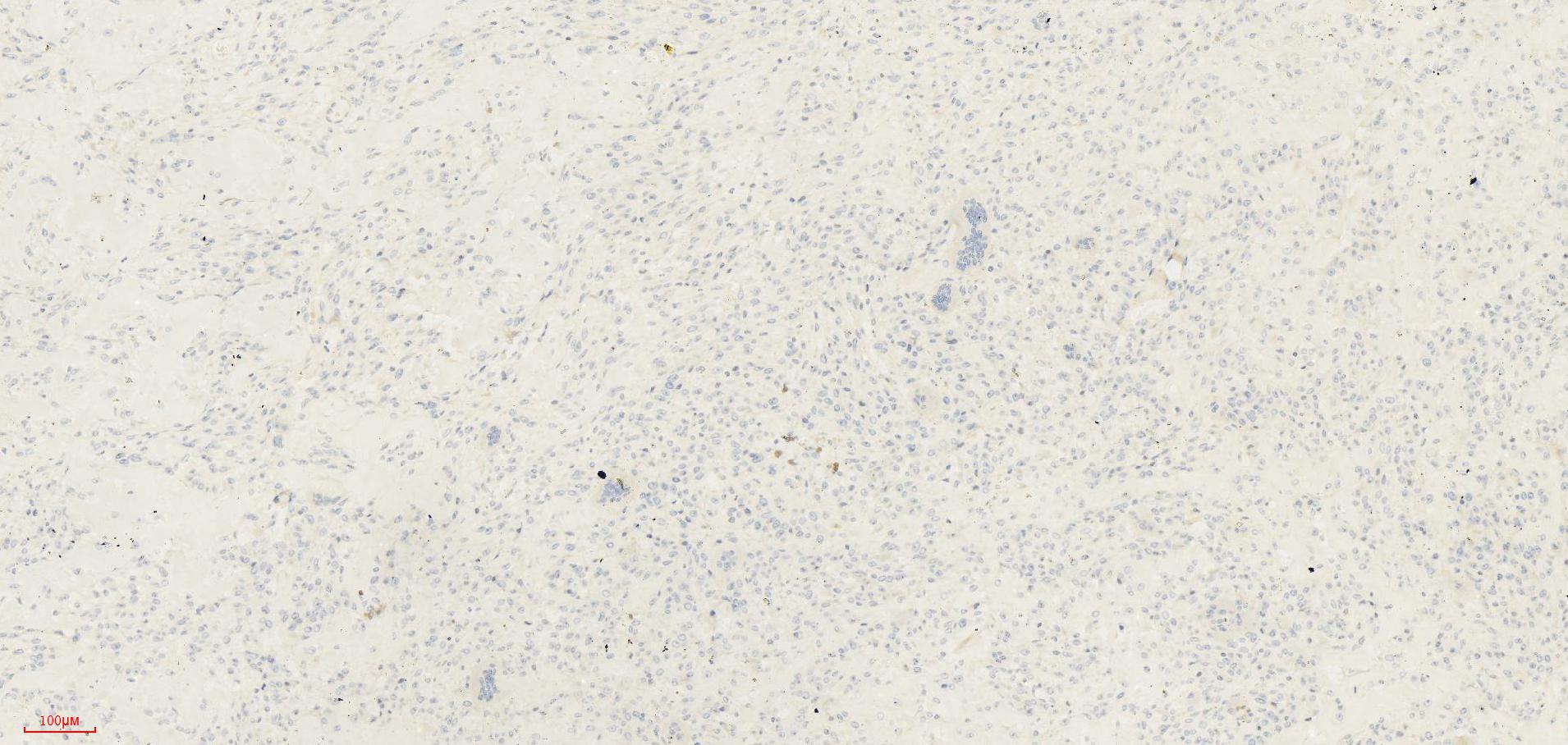

Supplement: Supplementary file 1 [file DataSheet_1.zip › Immunohistochemical analysis/τùàτÉå/ck-l 10X.jpg]

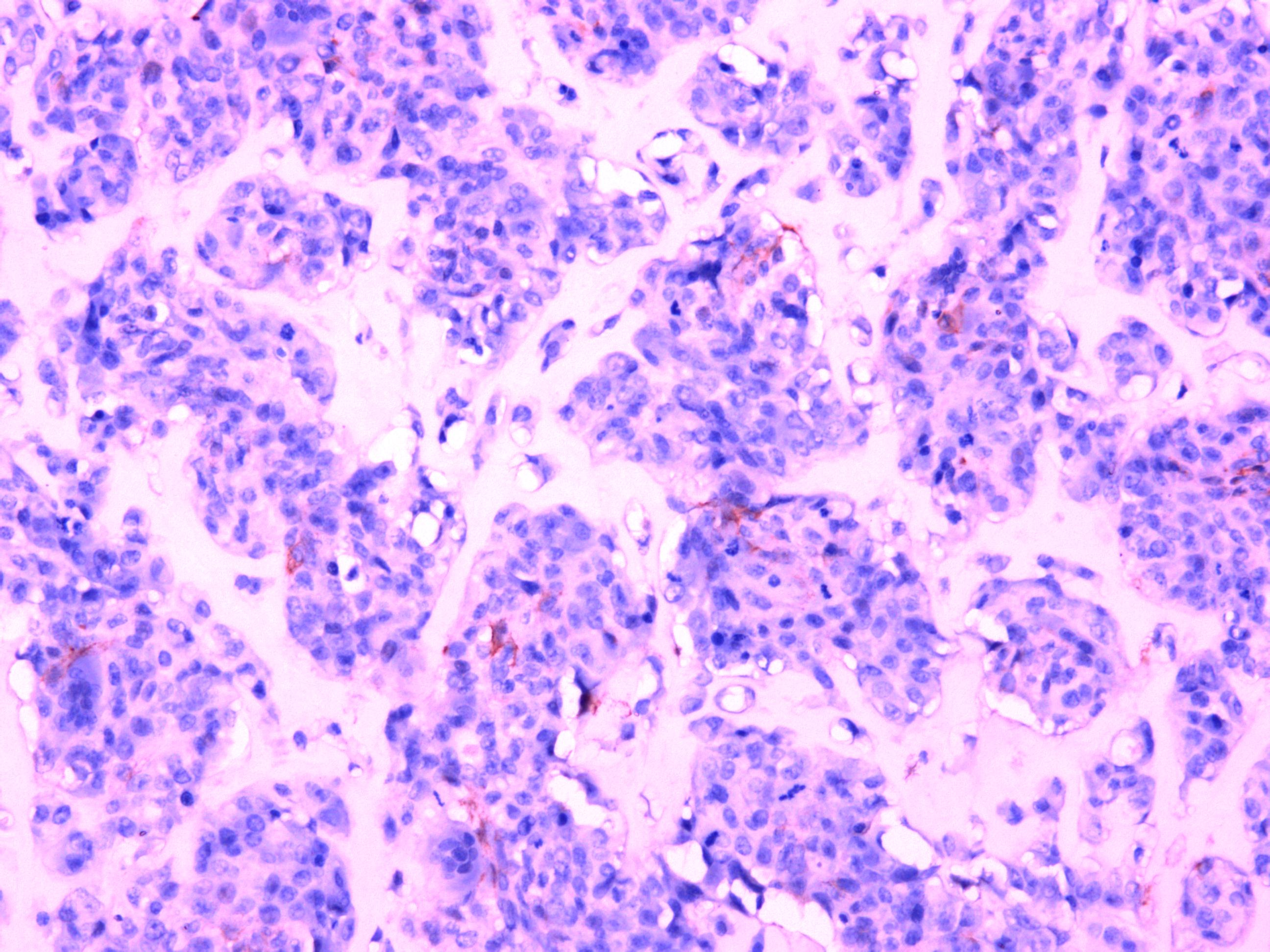

Supplement: Supplementary file 1 [file DataSheet_1.zip › Immunohistochemical analysis/τùàτÉå/CD10 200-4.JPG]

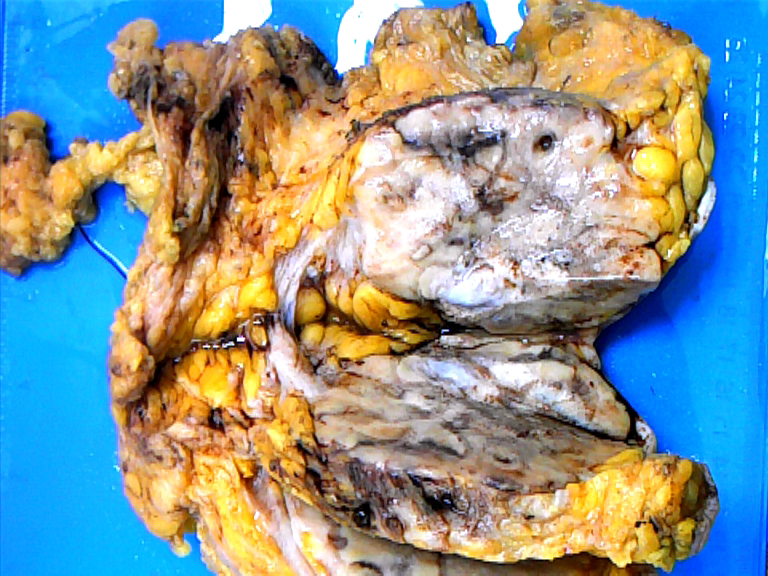

Supplement: Supplementary file 1 [file DataSheet_1.zip › Immunohistochemical analysis/τùàτÉå/1611811.bmp]

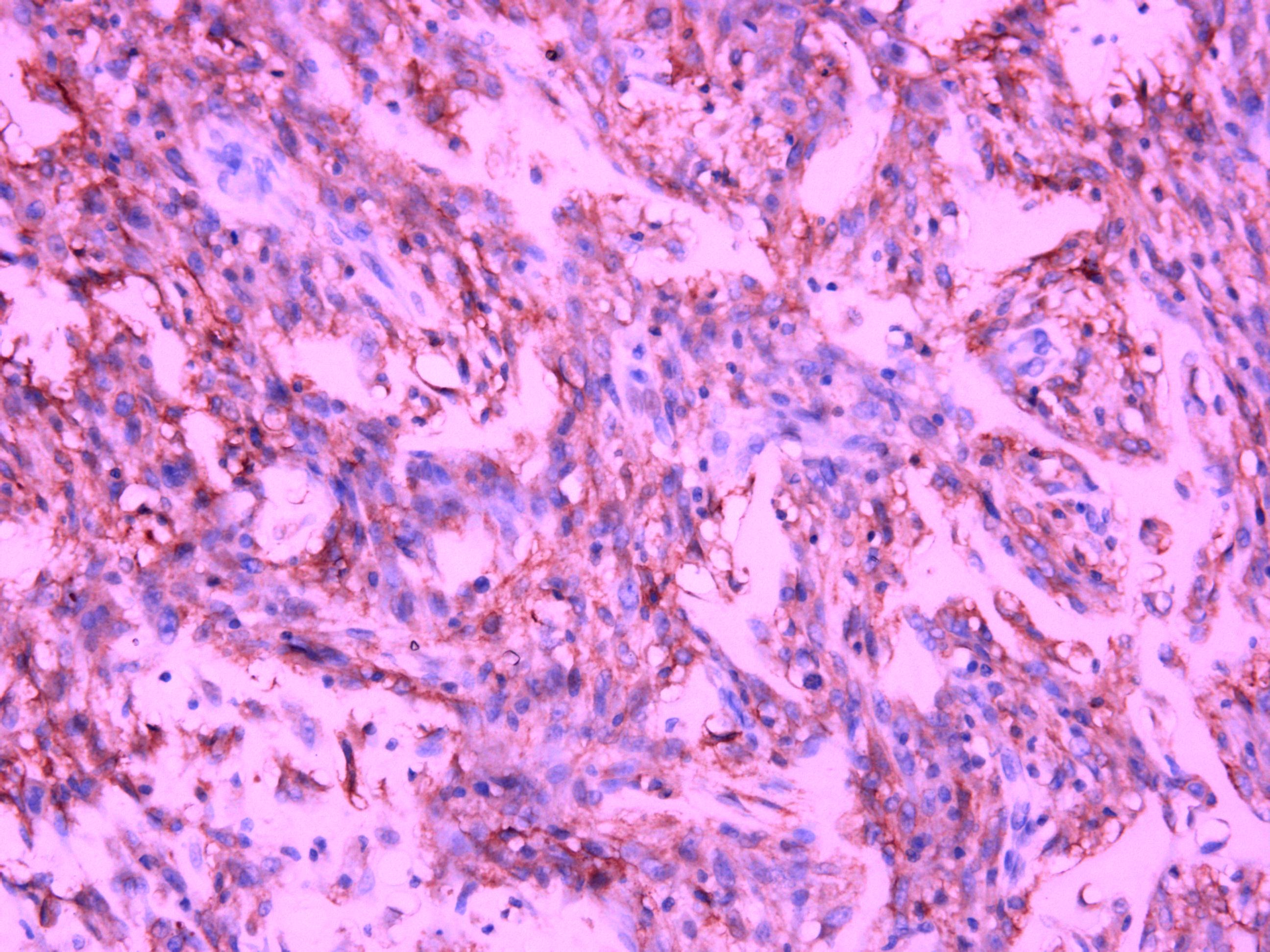

Supplement: Supplementary file 1 [file DataSheet_1.zip › Immunohistochemical analysis/τùàτÉå/CD10 200-1.JPG]

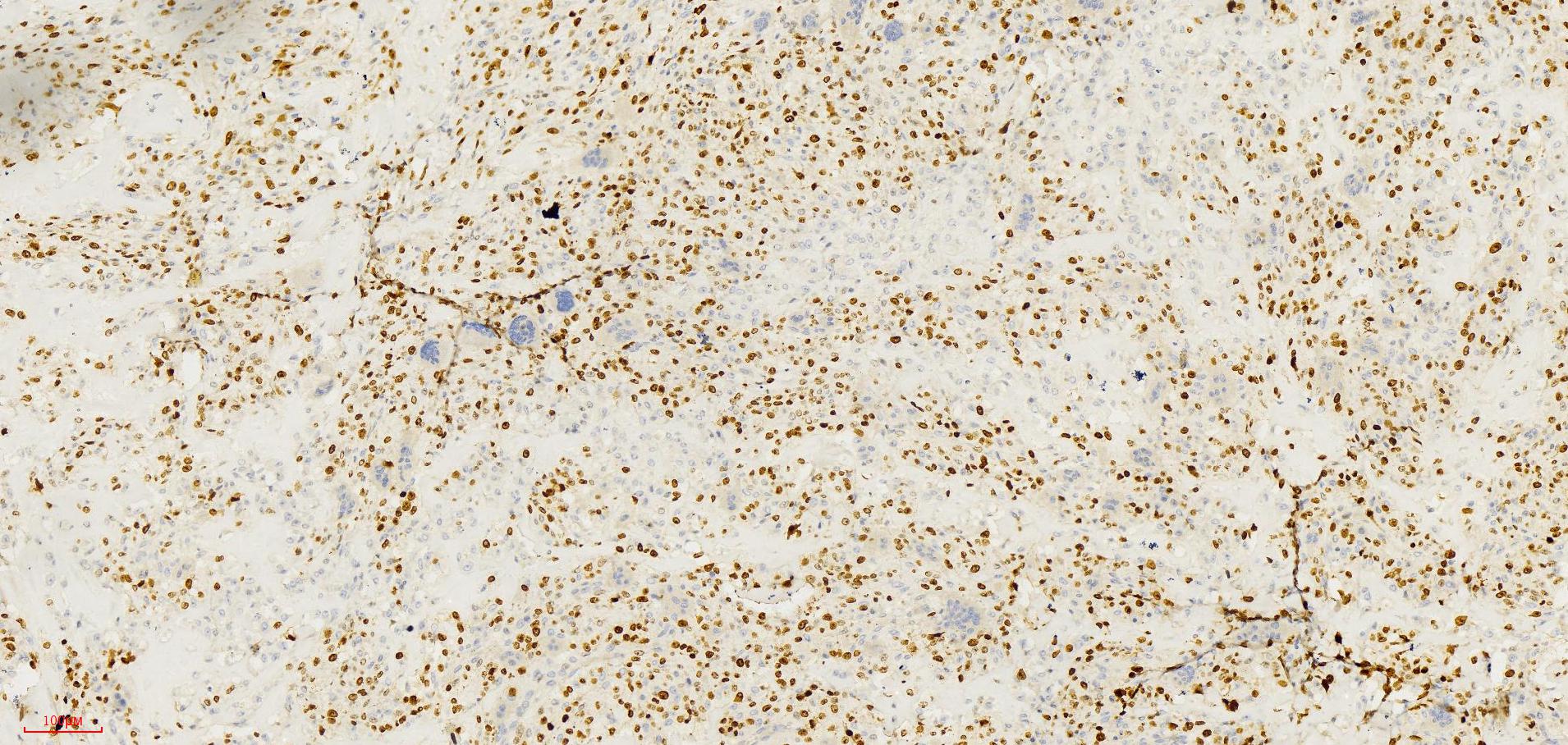

Supplement: Supplementary file 1 [file DataSheet_1.zip › Immunohistochemical analysis/τùàτÉå/ki67 10X.jpg]

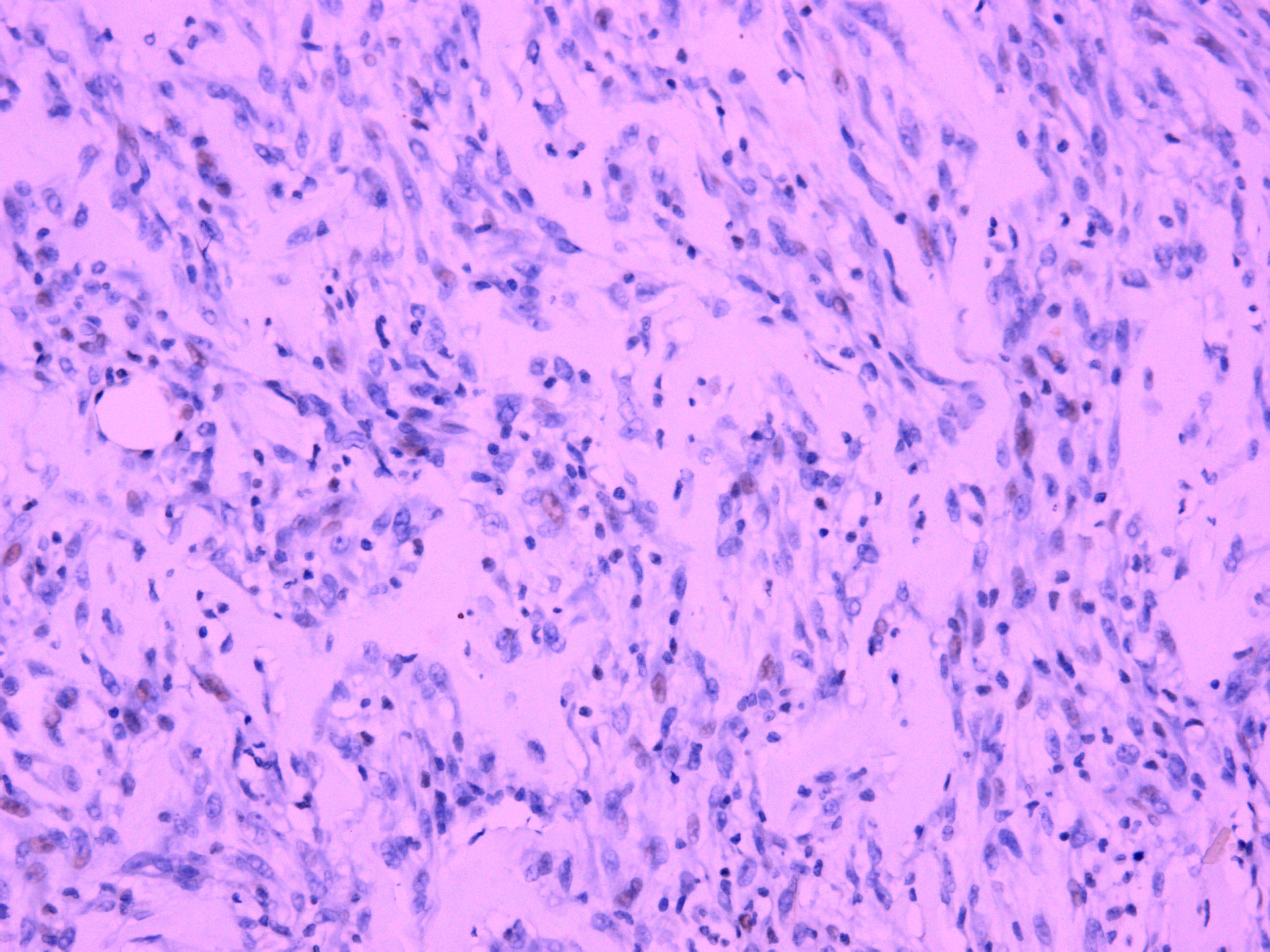

Supplement: Supplementary file 1 [file DataSheet_1.zip › Immunohistochemical analysis/τùàτÉå/ck56 200 .JPG]

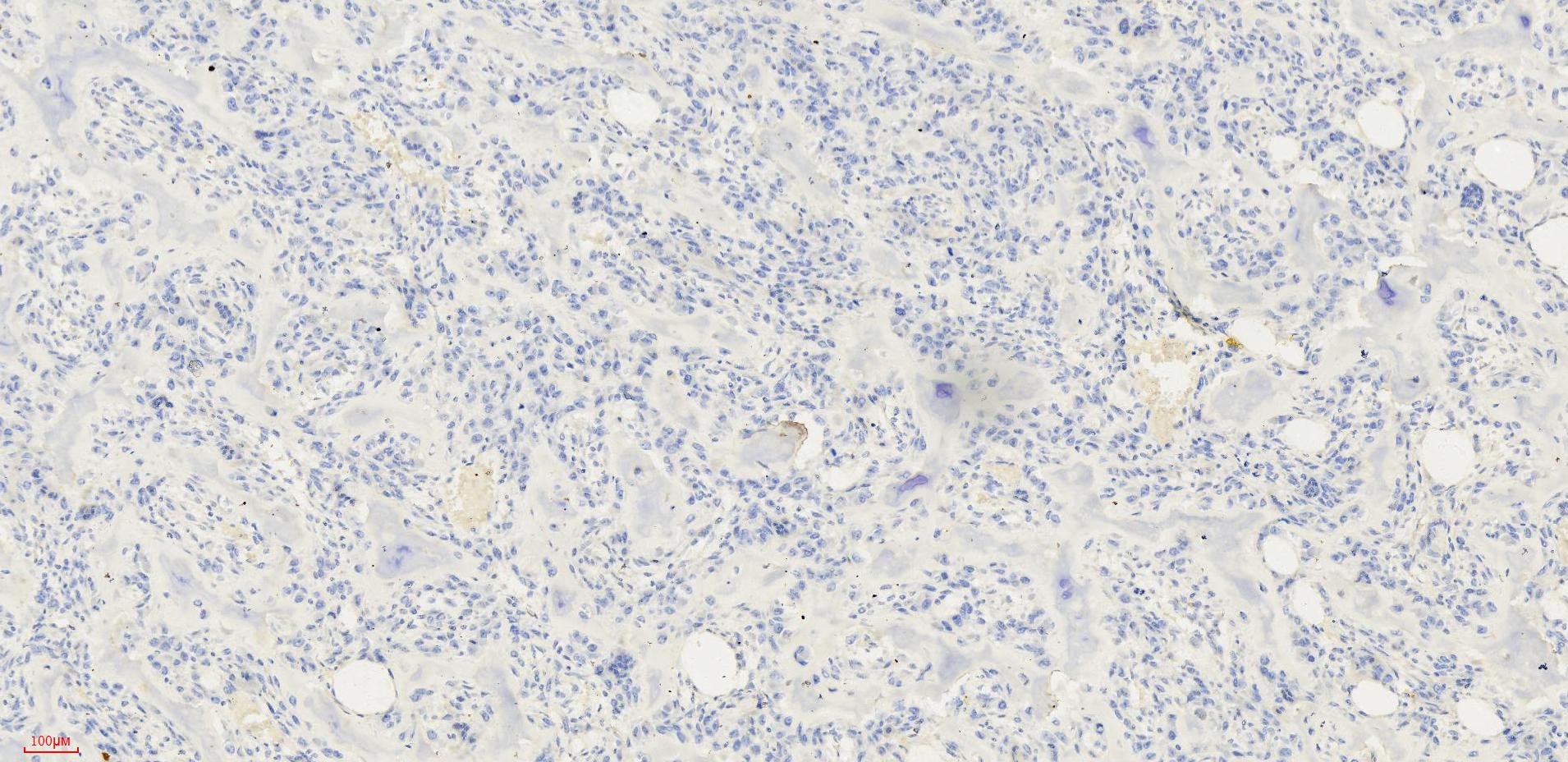

Supplement: Supplementary file 1 [file DataSheet_1.zip › Immunohistochemical analysis/τùàτÉå/ck-pan 10X.jpg]

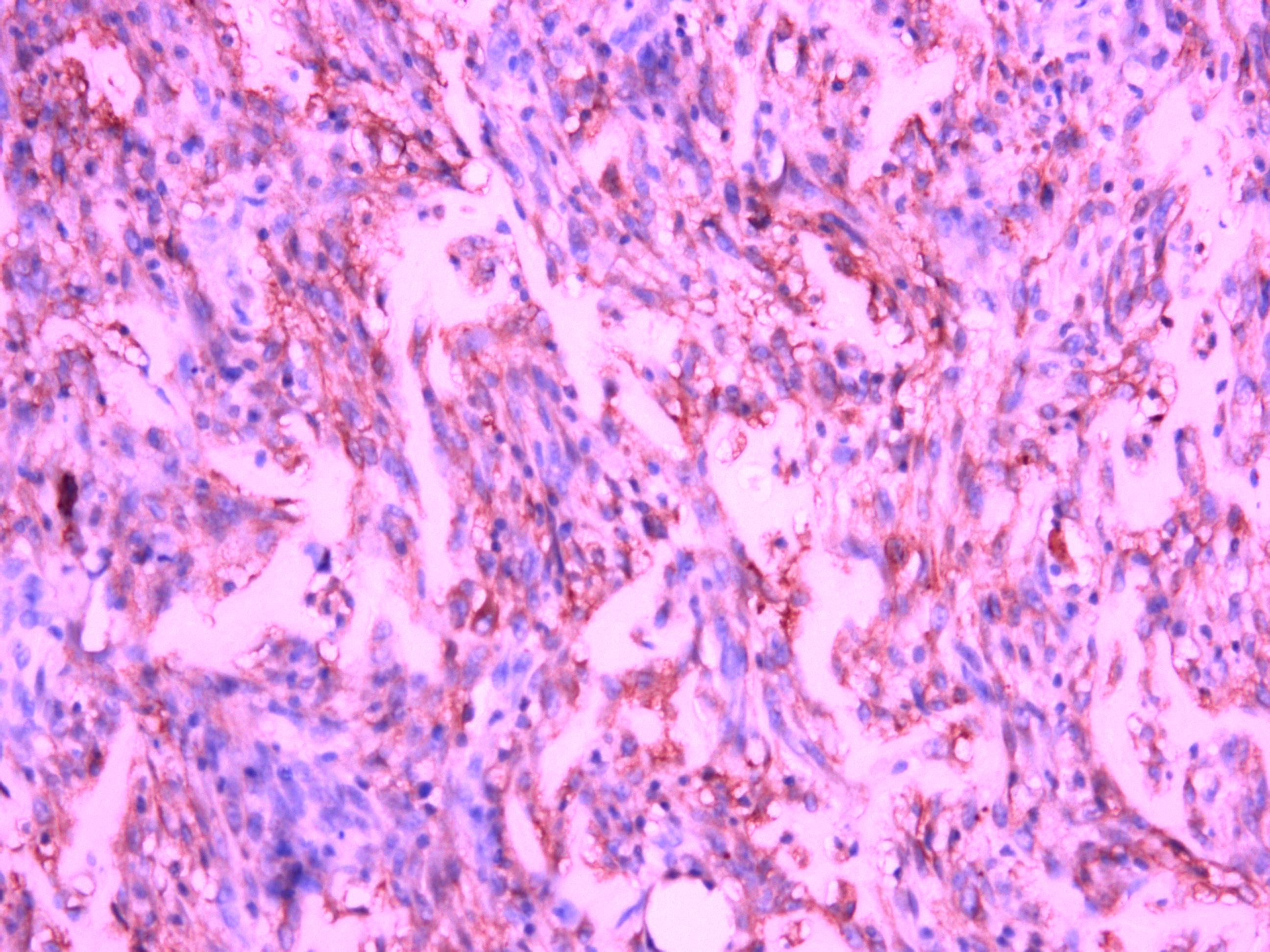

Supplement: Supplementary file 1 [file DataSheet_1.zip › Immunohistochemical analysis/τùàτÉå/CD10 200-2.JPG]

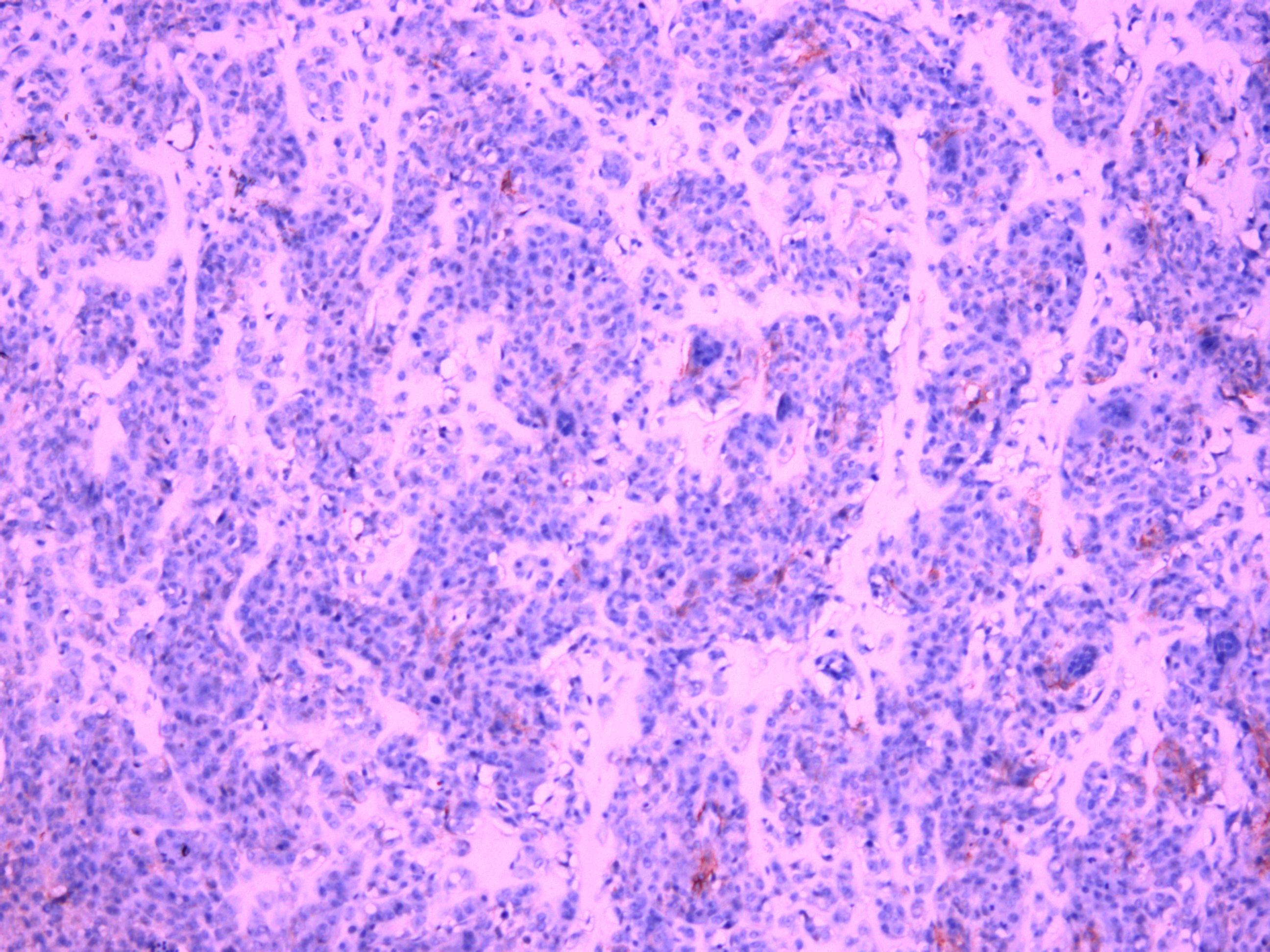

Supplement: Supplementary file 1 [file DataSheet_1.zip › Immunohistochemical analysis/τùàτÉå/CD10 200-3.JPG]

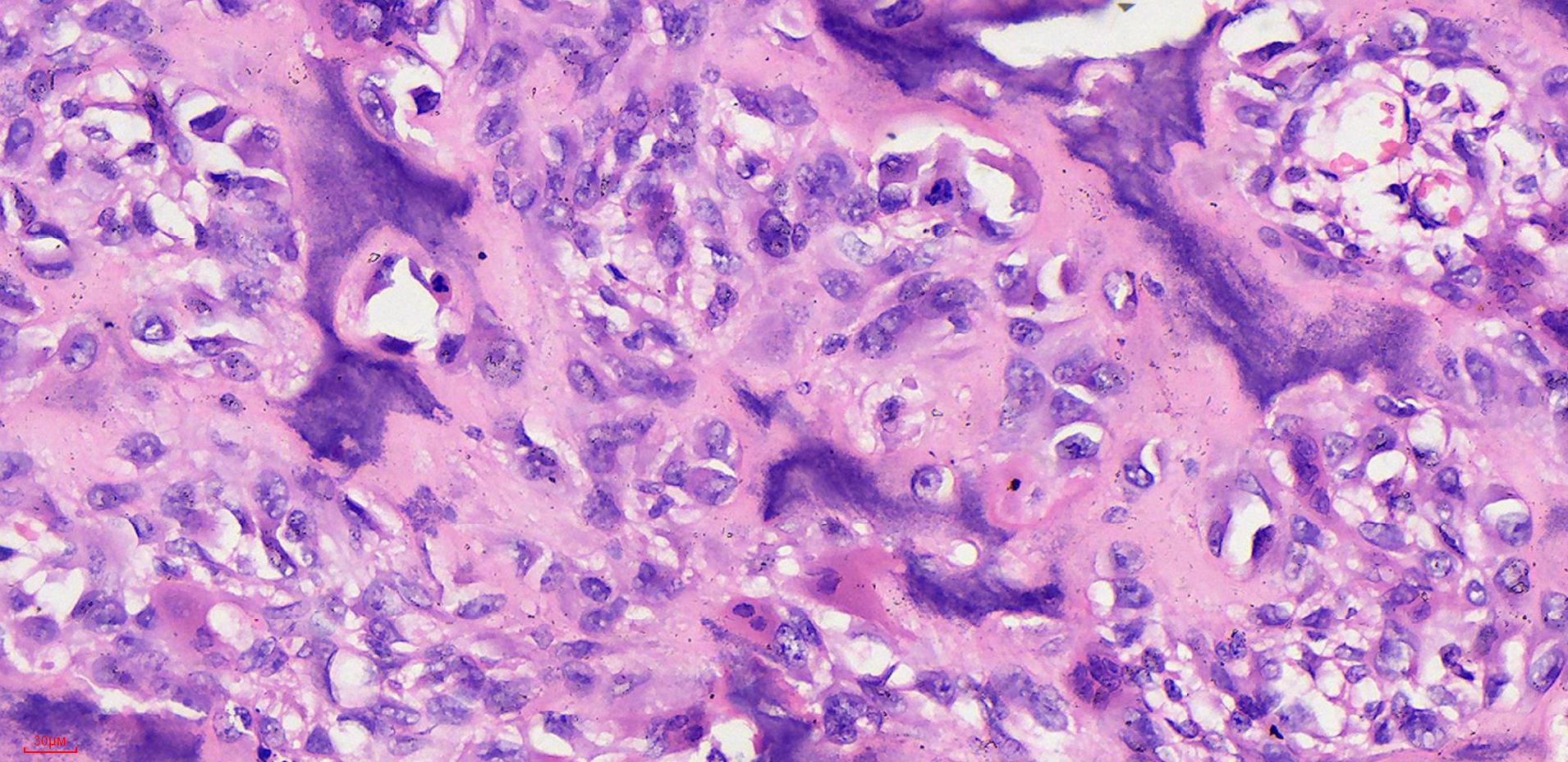

Supplement: Supplementary file 1 [file DataSheet_1.zip › Immunohistochemical analysis/τùàτÉå/HE 40X.jpg]
